# Insights into the venom composition and evolution of an endoparasitoid wasp by combining proteomic and transcriptomic analyses

**DOI:** 10.1038/srep19604

**Published:** 2016-01-25

**Authors:** Zhichao Yan, Qi Fang, Lei Wang, Jinding Liu, Yu Zhu, Fei Wang, Fei Li, John H. Werren, Gongyin Ye

**Affiliations:** 1 State Key Laboratory of Rice Biology & Ministry of Agriculture Key Laboratory of Agricultural Entomology, Institute of Insect Sciences, Zhejiang University, Hangzhou 310058, China; 2 Department of Entomology, College of Plant Protection, Nanjing Agricultural University, Nanjing 210095, China; 3 Department of Biology, University of Rochester, Rochester, NY 14627, USA

## Abstract

Parasitoid wasps are abundant and diverse hymenopteran insects that lay their eggs into the internal body (endoparasitoid) or on the external surface (ectoparasitoid) of their hosts. To make a more conducive environment for the wasps’ young, both ecto- and endoparasitoids inject venoms into the host to modulate host immunity, metabolism and development. Endoparasitoids have evolved from ectoparasitoids independently in different hymenopteran lineages. *Pteromalus puparum*, a pupal endoparasitoid of various butterflies, represents a relatively recent evolution of endoparasitism within pteromalids. Using a combination of transcriptomic and proteomic approaches, we have identified 70 putative venom proteins in *P. puparum*. Most of them show higher similarity to venom proteins from the related ectoparasitoid *Nasonia vitripennis* than from other more distantly related endoparasitoids. In addition, 13 venom proteins are similar to venoms of distantly related endoparasitoids but have no detectable venom matches in *Nasonia*. These venom proteins may have a role in adaptation to endoparasitism. Overall, these results lay the groundwork for more detailed studies of venom function and adaptation to the endoparasitic lifestyle.

Parasitoid wasps, being invaluable in classical and augmentative biological control of various insect pests, are among the most abundant and diverse insects on earth^1^. They have two basic lifestyles. Endoparasitoids lay their eggs into internal body of the host, whereas ectoparasitoids lay on the external surface of their hosts^1^,^2^. Parasitoids also injected substances into the host to ensure the successful parasitism and facilitate the successful development of their offspring, which can include venom, polydnaviruses (PDVs), virus-like particles (VLPs), ovarian fluids and teratocytes. The effects of these components depend largely on the parasitic life strategy^3^,^4^. Venoms from ectoparasitoids often induce a long-term paralysis to immobilize hosts, block their development following parasitism and also regulate their metabolism and immunity^5^,^6^. On the other hand, endoparasitoid venoms are mainly involved in temporary paralysis, host regulation by suppressing immune responses, delaying or arresting host development and synergizing the effects of PDVs/VLPs in some host-endoparasitoid systems^3^,^4^,^7^.

Venoms in most animals are involved in predation and/or defense^4^. They have been recognized as a rich source of biological active compounds^8^. In particular, venoms from cone snails^9^, snakes^10^, scorpions^11^, spiders^12^,^13^ and bees^14^ have received a great deal of attention. Intensive investigations have been done in these species to identify and characterize venom proteins with medical values by combining of transcriptomic, proteomic and peptidomic techniques. With an estimated number of species up to 600,000, parasitoid wasps account for around 75% of the described Hymenoptera and 10–20% of all insect species^15^, representing a group that dwarfs the set of venomous animals mentioned above. Venoms from parasitic Hymenoptera therefore could be an underestimated sources of valuable compounds that have potential use in pest control and pharmacy^16^,^17^. A few studies have been conducted on the compositions of several parasitoid venoms^18^,^19^,^20^,^21^,^22^,^23^,^24^,^25^,^26^,^27^. However, compared to their diversity, little is known about the composition, function and evolutionary relationship of different parasitoid venoms^3^,^4^.

*Pteromalus puparum* is a pupal endoparasitoid wasp that parasitizes a number of butterflies including the agricultural pest small cabbage white butterfly, *Pieris rapae*^28^. *Pteromalus puparum* belongs to the same subfamily Pteromalinae as the model parasitoid wasp *Nasonia vitripennis*. But, in contrast, *N. vitripennis* is an ectoparasitoid that parasitizes the pupae of various flies^29^. There are a number of independent origins of endoparasitoids evolving from ectoparasitoids, including in braconids, ichneumonids, chrysidoids, chalcidoids, ceraphronoids, evanioids and so on^30^. *P. puparum* represents a relatively recent evolution of endoparasitism within the subfamily Pteromalinae, and thus may provide a good model for comparative studies with *N. vitripennis* to better understand the differences and evolutionary relationship between endo- and ectoparasitoids.

Similar to *N. vitripennis*, venom from *P. puparum* is considered to be the major maternal factor that alters both the host immunity and physiology to facilitate the development of progenies^28^,^31^. No other virulence factors such as PDV, VLP and teratocyte, have been found in *P. puparum* so far. Our previous studies showed that *P. puparum* venom could inhibit both the cellular and humoral immunity of host, and regulate host development and metabolism^28^,^32^,^33^,^34^.

In this study, we investigated the *P. puparum* venom composition by combining both transcriptomic and proteomic approaches. Also taking differential expression and signal peptide analysis into consideration, we finally identified 70 venom gland differentially expressed secretory proteins as putative venom proteins in *P. puparum*. The results will help us to study the mode actions of these venom proteins, and to better understand the evolution of venoms among Hymenopteran parasitoids.

## Results

### Assembly and analyses of *P. puparum* transcriptome

**Figure 1:**
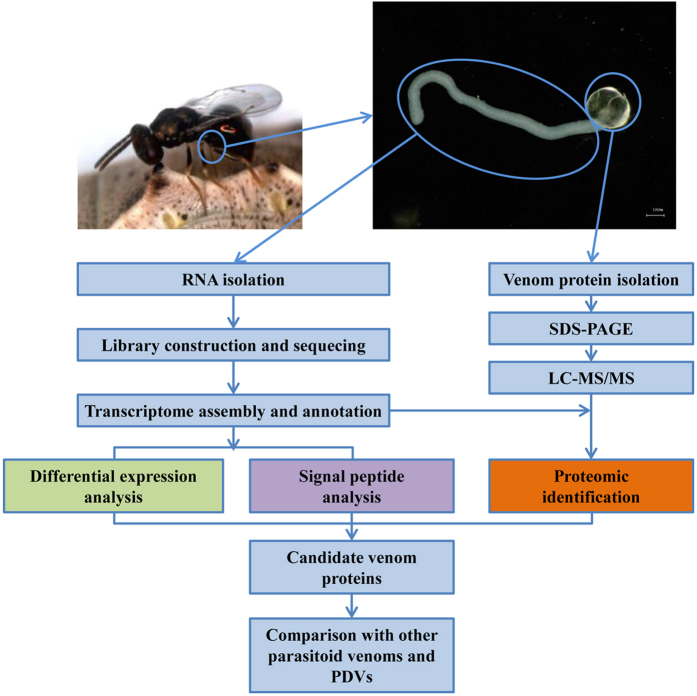
Schematic representation of combined proteomic and transcriptomic analyses to identify putative venom proteins in *Pteromalus puparum*.

Three cDNA libraries were separately constructed and sequenced for transcriptome assembly: whole female adults, venom glands and female body carcasses without venom apparatus. Then all the raw data was filtered and *de novo* assembled to create a *P. puparum* transcriptome (Fig. 1). Assembly statistics showed that the N50 was 2226 bp, and N80 was 825 bp (Table 1). Unigene represents a set of transcripts from the same transcription locus. Here the longest copy of redundant transcripts was regarded as a unigene. Finally, 39,738 unigenes that represented 55,958 transcripts were obtained (supplementary Figure S1). Among all unigenes, 43.73% (17,379) unigenes got matches in the nr database using blastx (e-value < 1e^−5^).

**Figure 2:**
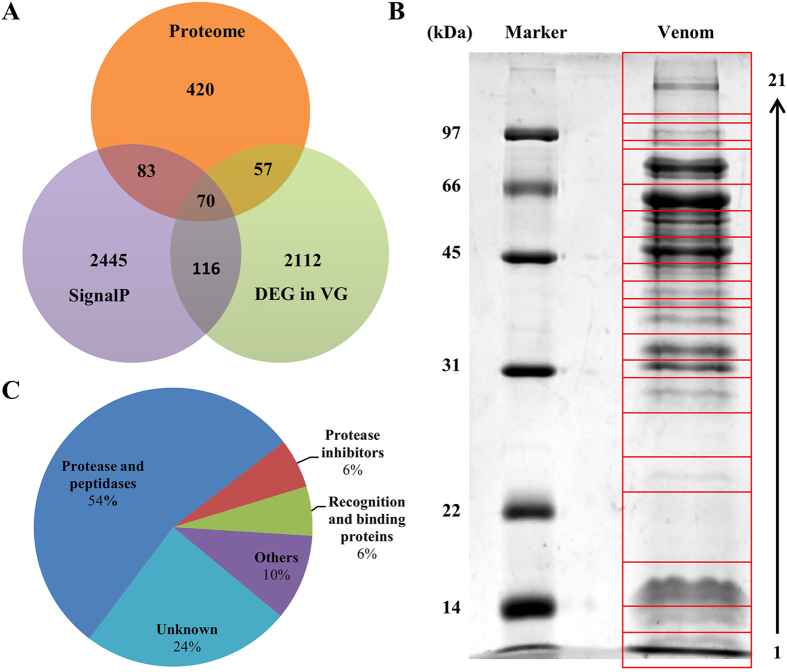
Identification of 70 putative venom proteins from *Pteromalus puparum*. (**A**) Venn diagram of putative venom proteins. The orange circle indicates unigenes which were identified by proteomic approach, the purple circle indicates unigenes with signal peptides, and the light green circle indicates unigenes which are differentially expressed in venom gland. (**B**) The SDS-PAGE (12%) analysis of venom protein. 21 gel slices are indicated by numbers on the right and shown in red boxes. The sizes and positions of molecular weight standards are indicated on the left. (**C**) The composition of venom proteins. DEG in VG: differentially expressed unigenes in venom gland; SignalP: signal peptide.

Venom gland cells from parasitoid wasps secrete venoms into the lumen of venom glands. Therefore, venom proteins are expected with secretory signal peptides in their amino acid sequences. For signal peptide analysis, transcripts with complete N terminal (subject start position of the best hit alignment = 1) were computationally translated into proteins and subjected to software SignalP. Simultaneously, their best hit sequences in nr database were also retrieved from NCBI as reference sequences for signal peptide analysis. The identity of the results by two different methods was 99% (Supplementary Table S1). Therefore, the signal peptide analysis of all unigenes was finally conducted using reference sequences. By this method, 2714 unigenes with signal peptides were identified in *P. puparum* combined transcriptome in total (Fig. 2A).

The expression levels of unigenes in both venom gland and carcass without venom apparatus were estimated by software eXpress^35^,^36^. To control false positive rate, the expression level cut-off was set as FPKM_VG (Venom gland) >10, and a venom gland to carcass expression ratio log_2_ (FPKM_VG/FPKM_Carcass) >1 and corrected P-value < 0.001 to define differentially expressed genes in venom gland. By this criterion, 2355 unigenes were identified differentially expressed in venom gland relative to carcass (whole female body minus the venom apparatus) (Fig. 2A).

### Identification of venom proteins by proteomic approach

For proteomic identification, venom proteins were separated by SDS-PAGE. Several apparent bands were observed with molecular masses ranging from less than 14 kDa to more than 97 kDa (Fig. 2B). And the most abundant band was a little below 66 kDa. The SDS-PAGE gel was cut into 21 slices as the graph showed (Fig. 2B). These slices were in-gel digested by trypsin and subjected to LC-MS/MS to identify the proteins. The database for proteomic research was generated by computationally translating transcriptomic sequences into proteins according to the blastx results. Finally, 630 unigenes were identified from the venom reservoir by this approach (Fig. 2A).

### Identification of putative venom proteins by combined analyses of transcriptomic and proteomic information

To identify a robust set of venom proteins, all data were analyzed under the assumption that venom proteins were secretory and differentially expressed in venom gland (Fig. 1). Combined transcriptomic and proteomic information, 70 unigenes were identified as secretory, differentially expressed in venom gland and confirmed by proteomic approach (Fig. 2A). In this study, these unigenes were defined as putative venom proteins for further analysis.

These 70 putative venom proteins were categorized into enzymes (38 records), protease inhibitors (4 records), recognition and binding proteins (4 records), others (6 records) and unknown (17 records) (Fig. 2C). The most abundant category (54%) is “enzymes”, which included esterase, serine proteases, metalloproteases, enzymes involved in DNA metabolism and so on, and the second (24%) is “unknown”. These proteins are described in more detail in supplementary file 1.

**Figure 3:**
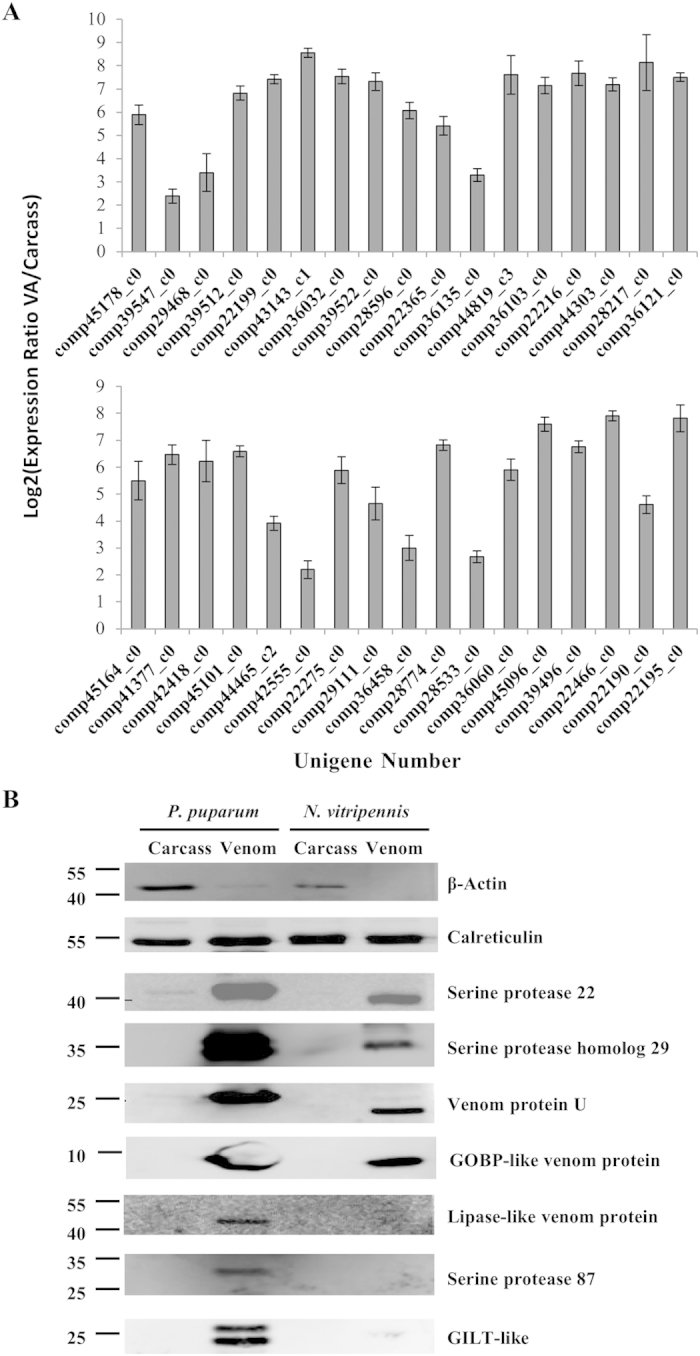
Verification of putative venom proteins by quantitative real-time PCR (qPCR) and Western blotting. (**A**) qPCR verification of selected putative venom proteins. The genes and primers used for these proteins are listed in Table S1. (**B**) Western blotting of venom proteins from *P. puparum* and *N. vitripennis*. β-Actin was used as housekeeping protein. The accession or unigene numbers of these venom proteins are as follows. calreticulin (GenBank: ACZ68113), serine protease 22 (comp44498_c3), serine protease homolog 29 (comp44055_c7), venom protein U (comp22466_c0), GOBP-like venom protein (comp39522_c0), lipase-like venom protein (comp28596_c0), serine protease 87 (comp43143_c1), GILT-like (comp36384_c0). VA: venom apparatus; Carcass: whole body of female adult without venom apparatus; GILT-like: gamma-interferon-inducible lysosomal thiol reductase-like.

Because the transcriptomic and proteomic sequencing are not replicated, gene expression in the venom gland was examined for 34 putative venom protein genes by qPCR, and 8 proteins were examined for their presence in venom reservoirs by Western blotting. All 34 tested venom protein genes were differentially expressed in venom gland related to carcass (Fig. 3A), all 8 proteins were confirmed by Western blotting using antibodies to the specific venom proteins (Fig. 3B). These results showed that the putative venom proteins set in this study is reliable.

### Similarity comparison of *P. puparum* venom proteins to *N. vitripennis* and other endoparasitoid venoms

**Figure 4:**
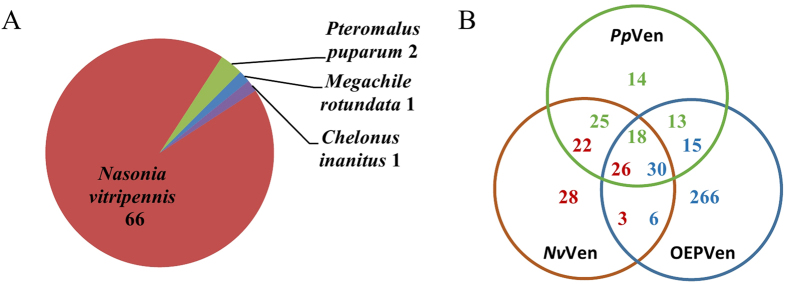
Comparison of venom proteins from *Pteromalus puparum* to *Nasonia vitripennis* and other endoparasitoid venoms. (**A**) Species distribution of the top BLASTX hit in the nr database for putative venom proteins from *P. puparum*. (**B**) The similarity comparison among *P. puparum*, *N. vitripennis* and other endoparasitoid venoms by BLASTP with a cutoff e-value ≤ 1e^−5^ and bit score ≥50. The green numbers indicate hits from *P. puparum* venom, the red numbers indicate hits from *N. vitripennis*, and the blue numbers indicate hits from other endoparasitoid venoms. As numbers of similar kind of proteins can be different in different venom sets, the hits from different venoms can be different in same category. *Pp*Ven: venom proteins from *P. puparum*. *Nv*Ven: venom proteins from *N. vitripennis*. OEPVen: venom proteins from other endoparasitoid wasps.

Comparisons of *P. puparum* venoms to venom and non-venom proteins in other parastitoids were investigated by three general methods. In our initial comparisons, we performed a blastx of *P. puparum* venom proteins against the nr database. Excluding self matches, the large majority (68 of 70) of *P. puparum* venom proteins have a best hit to proteins from the ectoparasitoid, *N. vitripennis* (Fig. 4A, Table 2). The remaining two gave best matches to a venom protein from the parasitoid *Chelonus inanitus* and a non-venom protein from the bee *Megachile rotundata*, respectively.

We next specifically compared *P. puparum* venom proteins to venoms reported in *N. vitripennis* and to our database of venoms from other endoparasitoids (OEP, see methods) using blastp (Supplementary Table S2). Of these 70 venoms, 48 (68.6%) gave better e-values and 46 (65.7%) gave better bit scores to *N. vitripennis* venom proteins than to OEP venoms. Therefore, most *P. puparum* venoms are more similar to *N. vitripennis* than OEP venoms (e-value Wilcoxon matched signs rank (WMSR) test, p = 0.009, Supplementary Figure S2, bit score WMSR test, p = 0.001, Supplementary Figure S3). This likely reflects the closer evolutionary relationship of the endoparasitoid *P. puparum* to the ectoparasitoid *N. vitripennis*, which are in the same subfamily Pteromalinae with similar morphology (Supplementary Figure S4), than to species in the OEP, which occur in other families and superfamilies of parasitoids (e.g. *Leptopilina boulardi*, *L. heterotoma*^21^, *Aphidius ervi*^20^, *Microplitis demolitor*^27^, *Microctonus* sp^24^. and *C. inanitus*^18^).

Using cut-off criteria (e-value ≤ 1e^−5^, bit score ≥ 50, see methods), we then assigned all proteins from the three venom data sets to shared and unshared categories (Fig. 4B). Based on the criteria, 14 venom proteins were found to be unique to *P. puparum*, while 25 were shared only with *N. vitripennis*, 13 were shared only with OEP, and 18 were shared among all three sets. *Pteromalus puparum* venom proteins are significantly more likely than are *N. vitripennis* venom proteins to show similarities only to OEP venoms (13/70 versus 3/79, two tailed fisher extract test, p = 0.006). Examples includ adenosine deaminase CECR1-like, protein lethal (2) essential for life-like, disulfide-isomerase A3-like, pancreatic triacylglycerol lipase-like, GILT-like, protein FAM151A-like. This set of venom proteins which only shared between *P. puparum* and OEP may have a role in the adaptation to endoparasitism.

Twenty-five venom proteins in *P. puparum* and 22 in *N. vitripennis* were shared in *P. puparum* and *N. vitripennis* venom only, and might be Pteromalinae venom specific. Some venom proteins which were previously reported as unique in *N. vitripennis* were also detected in *P. puparum* venom, including venom protein D, G, J, L, O, U and Z. Eighteen venoms were shared among all three data sets, and might present a core of venom proteins in parasitoid wasps, including venom allergen, calreticulin, serine protease, acid phosphatase, glucose dehydrogenase, gamma-glutamyltranspeptidase and so on (Supplementary Table S2).

### Test of *P. puparium* venom antibodies against *N. vitripennis* venom

Antibodies against *P. puparum* venom proteins were tested on *N. vitripennis* venom to see whether they could cross detect *N. vitripennis* venom proteins. Antibodies against *P. puparum* calreticulin, GOBP-like venom protein, venom protein U, serine protease 22 and serine protease homolog 29 could also cross detect the venom proteins in *N. vitripennis* (Fig. 3B). The results support the view that similar proteins are present in *N. vitripennis* venom and share antigenic similarities. Western blotting results also showed several venom proteins were not detected in *N. vitripennis* venom by the antibodies against *P. puparum* venom proteins (Fig. 3B). GILT-like protein was absent in the venom set of *N. vitripennis*, and as expected, couldn’t be cross detected in *N. vitripennis* venom by antibody against *P. puparum* GILT-like protein. And antibodies against *P. puparum* lipase-like venom protein and serine protease 87 also failed to cross detect the venom proteins in *N. vitripennis*. These failures might be caused by the divergence of antigens between *P. puparum* and *N. vitripennis* venom proteins, which could be sequence and/or modification differences.

## Discussion

Using high throughput RNA-sequencing technology, we first assembled the transcriptome of *P. puparum*, which is a pupal endoparasitoid. Differential expression analysis and signal peptide analysis were conducted to search the differentially expressed secretory proteins in venom gland. In parallel, we used the shotgun proteomic approach to analyze the composition of venom. Combined the venom proteomic data with the transcriptomic information, we finally identified a robust set of putative venom proteins.

In this study, we assumed that venom proteins from *P. puparum* were secretory and differentially expressed in venom gland. However, proteins that were not differentially expressed in venom gland could not be totally excluded, as venom proteins, such as heat shock proteins and arginine kinases that are commonly found in parasitoid venoms. In some extreme cases, like *L. boulardi*^21^, venom proteins were even not specifically expressed in the venom gland. In *P. puparum*, most venom proteins are likely to be differentially expressed in the venom gland, as confirmed by qPCR in this study. In addition, many unigenes (116) from the whole body transcriptome encoded secretory proteins and were significantly more highly expressed in venom gland, but not identified by the proteomic approach. These proteins could just have local functions in the venom gland or have been missed by the proteomic approach, especially for small peptides which may not be retained by SDS-PAGE and are easy to be missed especially when there was a lack of genomic information.

Despite the rigorous filtering, the venom composition of *P. puparum* is still found to be quite complex. It is reasonable to believe that parasitoid venoms are much more complex than venoms from social Hymentoptera^23^. The parasitoid venoms must target immunity, development, metabolism and sometimes even the host nervous system to ensure successful parasitism^4^,^23^. This is quite different from the function of venoms from social Hymenoptera, which are mainly used for predation and defense.

*Pteromalus puparum* evolved endoparasitism from an ectoparasitoid ancestor relatively recently within the pteromalids. In the subfamily Pteromalinae, the majority of species are ectoparasitoids, such as *Urolepis rufipes*^37^, *Trichomalopsis sarcophagae*^38^, *Muscidifurax raptor*^39^, *Nasonia* and so on. There are also several ectoparasitoid wasps in the genus Pteromalus. For example, both *P. cerealellae*^40^ and *P. sequester*^41^ are solitary ectoparasitoids of larvae of Coleoptera. Moreover, according to the phylogenetic analysis and substitution rate results of calreticulin from parasitoid wasps, *P. puparum* and *N. vitripennis* has a relatively small evolutionary distance (supplementary Figure S5, Table S3). The evolutionary distance between *P. puparum* and *N. vitripennis* is even smaller than that between *L. boulardi* and *L. heterotoma*, which are in the same genus and have been intensively compared^25^,^42^. Thus, *P. puparum* and *N. vitripenis* provide a good model for comparative studies between endo- and ectoparasitoids, and particularly to the evolutionary changes that occur when endoparasitism evolves from ectoparasitism.

As expected, most of (68/70) the identified venom proteins from the endoparasitoid *P. puparum* had significant similarities with proteins from the ectoparasitoid wasp *N. vitripennis*, which belongs to the same subfamily (Pteromalinae). Moreover, most of *P. puparum* venom proteins showed higher similarities to venom proteins from *N. vitripennis* rather than to other reported endoparasitoids. All these results are consistent with the fact that these endoparasitoids have different independent origins from ectoparasitoids^30^.

Although endoparasitoid wasps have different independent evolutionary origins, convergent recruiting of some similar proteins could still be expected. Strikingly, several *P. puparum* venoms are only shared with other reported endoparasitoids, and not present in venoms of its closest sequenced relative, *N. vitripennis*, which is an ectoparasitoid. These venom proteins may have a role in the adaptation to endoparasitism. However, it is also possible that this pattern is caused by incomplete characterization of the venom repertoire of these species. Further investigation is therefore needed.

The development of the stinger and venoms in Hymenoptera had a single origin^30^. So it is expected that parasitoid wasps might contain some ancestral venom proteins. Venom antigen 5 is an example of such conservation as it is found from social Hymenoptera to parasitoid wasps (Supplementary Figure S6). In addition, different proteins have been recruited into venom for similar functions in different parasitoid wasps. These include superoxide dismutase (SOD) from *Leptopilina boulardi* and *L. heterotoma*^43^ and unrelated peptide Vn 4.6 from *Cotesia rubecula*^44^ which are known to inhibit the pro-phenoloxidase activation. A second example is RhoGAP (Ras homologous GTPase activating protein) from *L. boulardi*^45^, VPr3 from *Pimpla hypochondriaca*^46^,^47^ and SERCA (sarco/endoplasmic reticulum calcium ATPase) from *Ganaspis sp*.1^48^, which are very different proteins, but all are known to alter the behavior of host hemocytes.

Taking into consideration the complexity and diversity of parasitic factors delivered to hosts (including venom, PDV and others), parasitoid wasps seem to be an untapped source of valuable molecules with agricultural and medical potential^16^,^17^. Of course, a lot of work on the compositions of parasitoid venom, functions and applications of individual venom proteins is still needed. The identification of venom composition from *P. puparum* in this current study is the basis for further detailed analyses of the functions of these venom proteins.

Our research revealed closer relationship of most *P. puparum* venom proteins to those from the pupal ectoparasitoid *N. vitripennis*, rather than to other reported endoparasitoid wasps. Thirteen *P. puparum* venom proteins show similarity to other endoparasitoid venoms but not to venom proteins of the more closely related ectoparasitoid *N. vitripennis*. These proteins are promising candidates for a functional role in the evolution of endopariasitism. These results will open the way to a better understanding of venom evolution in the transition from ectoparasitoids to endoparasitoids.

## Methods

### Insect rearing

Laboratory cultures of *P. puparum* and *N. vitripennis* were maintained at 25 °C with a photoperiod of 14: 10 h (light: dark) as described previously^28^,^31^ and used in all experiments. Once emerged, the wasp females were collected and held in glass containers, fed *ad lib* on 20% (v/v) honey solution to lengthen life span.

### Venom gland preparation and RNA isolation

Mated female wasps aged 0–7 days were anaesthetized in −70 °C refrigerator for 5 min, and dissected in Ringer’s saline (KCl 182 mM; NaCl 46 mM; CaCl_2_ 3 mM; Tris-HCl 10 mM) with 1 unit/μl RNAase inhibitor (TOYOBO, Osaka, Japan) on the ice plate under a stereoscope (Olympus). Venom glands and carcasses without venom apparatus were collected into Trizol reagent (Invitrogen, USA), respectively. The total RNA was extracted using Trizol reagent according to the manufacture’s protocol.

### Construction and sequencing of cDNA library

The construction and sequencing of cDNA library were done by Beijing Genomics Institute (BGI, Shenzhen, China). Briefly, the isolated RNA was purified using Sera-mag Magnetic Oligo (dT) beads (Illumina), then transcribed using N6 primers followed by synthesis of second cDNA strand. After end pair processing and ligation of adaptor, RNA was amplified by PCR and purified using QIAquick Gel extraction Kit (Qiagen, Germany). The cDNA library of whole female adult was sequenced on Illumina Hiseq 2000 with paired-end reads of 100 bp. The cDNA libraries of venom gland and carcass were sequenced on 1G Illumina Genome Analyzer (Illumina, San Diego, USA) with paired-end reads of 75 bp.

### Analysis of transcriptomic data

The transcriptomic raw data was assembled using Trinity v2013-02-16^49^. All unigenes were annotated by blastx search against NCBI nr database (March, 2013) with a cutoff of 1e^−5^. The expression level was estimated by software eXpress v1.3.3^35^,^36^. Differential expression analysis between venom gland and carcass was performed using the R package DEGSeq v1.2.2^50^. The p-values were adjusted using the Benjamini & Hochberg method. Corrected p-value < 0.001, log_2_ (FPKM_VG/FPKM_Carcass) >1 and FPKM_VG (Venom gland) >10 were set as the threshold for significantly differential expression in venom gland. Presence of signal peptides was analyzed by software SignalP 4.1^51^. The putative venom unigenes were manually checked using blastx on NCBI website, and categorized into enzymes, protease inhibitors, recognition and binding proteins, others and unknown based on their blast results and domain information.

### Comparison of *P. puparum* venom proteins to *N. vitripennis* and other endoparasitoid venoms

For similarity comparison, blastp were performed between three different venom data sets, *P. puparum* venom, *N. vitripennis* venom^23^ and a database of other endoparasitoid venoms generated here. The other endoparasitoid venom database was manually generated, including venom proteins from *Leptopilina boulardi*, *L. heterotoma*^21^, *Aphidius ervi*^20^, *Microplitis demolitor*^27^, *Microctonus* sp^24^. and *Chelonus inanitus*^18^. All the nucleotide acid sequences from *P. puparum* venom and other endoparasitoid venoms were translated into proteins by OrfPredictor^40^ (http://proteomics.ysu.edu/tools/OrfPredictor.html). And venom proteins in other endoparasitoid venom database were further clustered by CD-HIT^41^ with sequence identity cutoff=0.5 (http://weizhong-lab.ucsd.edu/cdhit_suite/cgi-bin/index.cgi?cmd=cd-hit) to remove redundancy. As bit score is independent on database size and more suitable than e-value for comparing similarity scores from different searches (http://www.ncbi.nlm.nih.gov/BLAST/tutorial/), we analyzed a range of bit scores to determine how these criteria affect similarity scores among the different venom protein sets (Supplementary Table S4). Criteria that incorporated a bit score criterion (e-value ≤ 1e^−5^, bit sore ≥ 50) was used for the further analyses.

Multiple amino acid sequence alignments were performed using MUSCLE v3.8^52^. Phylogenetic analysis was conducted by MEGA 5 using maximum likelihood algorithm^53^. Pairwise substitution rates were calculated by CodeML in PAML v4.8^54^.

### Extraction of venom proteins

Mated female wasps aged 0–7 days of *P. puparum* and *N. vitripennis*, were anaesthetized in −70 °C refrigerator for 5 min as mentioned above, and then dissected in Ringer’s saline (KCl 182 mM; NaCl 46 mM; CaCl_2_ 3 mM; Tris-HCl 10 mM) with 1 mM phenylmethanesulfonyl fluoride (PMSF) (Sigma, St. Louis, MO) on the ice plate under a stereoscope (Olympus). The venom reservoirs were washed for several times, and then transferred to an Eppendorf tube. After centrifugation at 16,000 g and 4 °C for 1 min, the supernatant was filtered with 0.22 μm Millipore filter and stored at −70 °C until use. The concentration of venom protein was determined using Bradford method^55^.

### Mass spectrometric venom protein identification by LC-MS/MS

*Pteromalus. puparum* venom sample containing 100 μg proteins dissolved in 20 μl rehydration solution (7 M urea, 2 M thiourea, 4% CHAPS, 0.5% Triton X-100, 65 mM DTT, 0.5% Bio-Lyte, and 0.001% bromophenol blue) were separated by SDS-PAGE and stained with Coomassie Brilliant Blue R-250 (Bio-Rad, USA). The gel was excised into 21 slices, depending on the molecular masses of protein bands. Each gel slice was digested by trypsin and lyophilized separately followed by 1DLC-LTQ-Velos (Thermo Finnigan, San Jose, CA). In this study, samples were desalted on Zorbax 300 SB-C18 (Agilent Technologies, Wilmington, DE) and then separated on a RP-C18 column (150 μm i.d., 150 mm length) (Column technology Inc., Fremont, CA). The buffer A was water with 0.1% formic acid, buffer B was 84% acetonitrile with 0.1% formic acid, and the gradient was from 4% buffer B to 50% buffer B in  1h. The charge-to-mass ratios of peptides and fractions of peptides were collected 20 times after every full scan. The resulting MS/MS spectra were searched against the translated *P. puparum* transcriptome using Sequest search algorithm^56^. Carbamidomethyl of cysteine and oxidation of methionine were set as fixed and variable modifications, respectively. Delta CN (≥0.1) and cross-correlation scores (Xcorr, one charge ≥1.9, two charges ≥2.2, three charges ≥3.75) were used to filter the peptide identification. This part was done by Shanghai Applied Protein Technology Co., Ltd (Shanghai, China).

### Quantitative real-time PCR (qPCR)

cDNA from venom glands and carcasses without venom apparatus was synthesized, respectively, using TransScript one-step gDNA Removal and cDNA Synthesis SuperMix (TransGen, China) with random primers. All the primer sequences (Supplementary Table S5) used were designed on website Primer 3^57^ and synthesized commercially (Sangon, Chnia). The PCR reaction was run in ABI 7500 Real Time PCR System (Applied Biosystems, Foster City, CA) using SsoFast EvaGreen Supermix with Low Rox (Bio-Rad, USA) according to the manufacture’s protocol. The cycling conditions for qPCR were as follows: enzyme activation at 95 °C for 30 sec, followed by 40 cycles with denaturation at 95 °C for 5 sec, annealing at 60 °C for 34 sec. Relative expression level of putative venom proteins was normalized to reference gene (18S rRNA) using 2^−ΔΔCT^ method^58^. Statistical analysis was performed using Student’s t test. Unigenes with log_2_(Expression ratio venom gland/carcass) >1 and p-values < 0.05 were considered differentially expressed in venom gland.

### Western blotting

The antibodies against different *P. puparum* venom proteins were prepared as previously described^59^. Recombinant venom protein GOBP was expressed in the pGEX-4T-2 vector with a GST tag, others were expressed in pET-28a vector with a His tag. The primary antibody against β-actin was bought commercially (Huabio, China). The venom and carcass proteins of *P. puparum* and *N. vitripennis* were separated by 12% SDS-PAGE, and then transferred to a polyvinylidene difluoride (PVDF) membrane (Bio-Rad, USA) using Mini-ProTEAN Tetra system (Bio-Rad, Hercules, CA) at 16 V for 16 h. The PVDF membrane was blocked and washed. Anti-venom protein antibodies (diluted from 1: 500 to 1: 2000, depending on the antibody) and anti-actin antibody (diluted 1: 5000) were respectively used as the primary antibody. And goat anti-rabbit IgG-horseradish peroxidase (HRP) conjugate (Sigmae Aldrich, Taufkirchen, Germany; diluted 1: 5000) was used as the secondary antibody. The PVDF membranes were detected using ECL Western Blotting Substrate (Promega, Madison, WI, USA) and imaged in Chemi Doc-It^TM^ 600 Imaging System (UVP, Cambridge, UK).

### Availability of supporting data

All RNA-seq raw data have been deposited at the NCBI Sequence Read Archive under accession number SRP055738. This Transcriptome Shotgun Assembly project has been deposited at GenBank under the accession GECT00000000. The version described in this paper is the first version, GECT01000000.

## Additional Information

**How to cite this article**: Yan, Z. *et al.* Insights into the venom composition and evolution of an endoparasitoid wasp by combining proteomic and transcriptomic analyses. *Sci. Rep.* **6**, 19604; doi: 10.1038/srep19604 (2016).

## Supplementary Material

Supplementary Information

Supplementary Tables

## Figures and Tables

**Table 1:** Overview of *Pteromalus puparum* transcriptome.

Number of reads from VG	29,540,102
Number of reads from Carcass	28,109,926
Number of reads from FA	27,216,094
Number of assembled transcripts	55958
Transcripts longer than N50	8740
The shortest length	201 bp
The longest length	15705 bp
N50	2226 bp
N80	825 bp
N20	4279 bp

The cDNA library of FA was sequenced on Illumina Hiseq 2000 with paired-end reads of 100 bp. The cDNA libraries of VG and Carcass were sequenced on 1G Illumina Genome Analyzer with paired-end reads of 75 bp.VG: venom gland; Carcass: whole body of female adult without venom apparatus; FA: whole body of female adult.

**Table 2:** Candidate venom proteins identified in *Pteromalus puparum*.

Genes	VG-FPKM	Carcass-FPKM	log2(VG-FPKM/Carcass-FPKM)	NR ID	NR Description
Proteases and peptidases					
comp40292_c0	64.12	24.91	1.36	XP_001604991.1	PREDICTED: chymotrypsin-1 [Nasonia vitripennis]
comp44498_c3	224.81	22.34	3.33	NP_001155043.1	serine protease 22 precursor [Nasonia vitripennis]
comp36113_c0	2008.26	2.67	9.56	NP_001155017.1	serine protease 33 precursor [Nasonia vitripennis]
comp29468_c0	1131.96	4.01	8.14	NP_001166090.1	serine protease 73 precursor [Nasonia vitripennis]
comp43143_c1	12010.35	34.87	8.43	NP_001166092.1	serine protease 87 precursor [Nasonia vitripennis]
comp43143_c3	320.51	1.59	7.66	NP_001166092.1	serine protease 87 precursor [Nasonia vitripennis]
comp44055_c7	7795.75	14.18	9.10	NP_001155016.1	serine protease homolog 29 precursor [Nasonia vitripennis]
comp36103_c0	3127.21	4.39	9.48	NP_001164348.1	serine protease precursor [Nasonia vitripennis]
comp40194_c0	353.14	0.62	9.16	XP_001600730.2	PREDICTED: blastula protease 10-like [Nasonia vitripennis]
comp29111_c0	373.03	2.54	7.20	XP_001604431.1	PREDICTED: A disintegrin and metalloproteinase with thrombospondin motifs 16-like [Nasonia vitripennis]
comp41685_c0	496.71	0.43	10.16	XP_001606746.2	PREDICTED: hypothetical protein LOC100123135 [Nasonia vitripennis]/region_name=“ZnMc”
comp6391_c0	11.58	0.00	∞	XP_001607602.1	PREDICTED: hypothetical protein LOC100123845 [Nasonia vitripennis]/region_name=“ZnMc”
comp44819_c3	1303.00	1.42	9.84	NP_001154991.1	lipase A-like precursor [Nasonia vitripennis]
comp28596_c0	1443.05	2.93	8.95	NP_001155039.1	lipase-like venom protein precursor [Nasonia vitripennis]
comp41786_c2	671.63	2.61	8.01	NP_001155039.1	lipase-like venom protein precursor [Nasonia vitripennis]
comp42555_c0	259.37	0.58	8.81	XP_003425033.1	PREDICTED: lipase member H-like [Nasonia vitripennis]
comp45112_c0	3395.22	6.89	8.94	XP_003425157.1	PREDICTED: pancreatic lipase-related protein 2-like [Nasonia vitripennis]
comp28462_c0	1339.53	3.39	8.63	XP_003426830.1	PREDICTED: pancreatic lipase-related protein 2-like [Nasonia vitripennis]
comp22275_c0	209.17	0.48	8.78	XP_003425157.1	PREDICTED: pancreatic lipase-related protein 2-like [Nasonia vitripennis]
comp36060_c0	1523.36	1.18	10.34	XP_003427888.1	PREDICTED: pancreatic triacylglycerol lipase-like [Nasonia vitripennis]
comp22302_c0	2085.48	2.72	9.58	XP_001605737.2	PREDICTED: hypothetical protein LOC100122136 [Nasonia vitripennis]/region_name=“Abhydro_lipase”
comp44469_c0	87.90	16.55	2.41	XP_001601350.2	PREDICTED: esterase E4 [Nasonia vitripennis]
comp43397_c2	588.20	1.07	9.10	XP_003427357.1	PREDICTED: venom acid phosphatase Acph-1-like [Nasonia vitripennis]
comp23069_c0	1158.10	6.40	7.50	XP_001605452.1	PREDICTED: venom acid phosphatase Acph-1-like isoform 1 [Nasonia vitripennis]
comp36032_c0	7780.99	16.12	8.91	ACA60733.1	venom acid phosphatase [Pteromalus puparum]
comp43694_c1	11.98	0.13	6.57	XP_003428033.1	PREDICTED: ribonuclease 1-like [Nasonia vitripennis]
comp28533_c0	1882.32	56.40	5.06	NP_001155172.1	inosine-uridine preferring nucleoside hydrolase-like precursor [Nasonia vitripennis]
comp42418_c0	3923.13	7.94	8.95	NP_001155087.1	endonuclease-like venom protein precursor [Nasonia vitripennis]
comp45389_c0	210.31	0.40	9.05	XP_003423840.1	PREDICTED: adenosine deaminase CECR1-like [Nasonia vitripennis]
comp28685_c0	155.45	25.07	2.63	NP_001153351.1	glucosamine (N-acetyl)-6-sulfatase precursor [Nasonia vitripennis]
comp22216_c0	6576.38	5.64	10.19	XP_001602184.1	PREDICTED: alpha-amylase 1-like [Nasonia vitripennis]
comp41097_c0	28.35	3.22	3.14	XP_003427944.1	PREDICTED: glucose dehydrogenase [acceptor]-like [Nasonia vitripennis]
comp45178_c0	607.76	0.77	9.62	XP_001604839.1	PREDICTED: gamma-glutamyltranspeptidase 1 [Nasonia vitripennis]
comp36384_c0	1065.20	92.54	3.52	XP_001606905.1	PREDICTED: gamma-interferon-inducible lysosomal thiol reductase-like [Nasonia vitripennis]
comp39547_c0	1377.55	6.52	7.72	XP_001607237.1	PREDICTED: kynurenine-oxoglutarate transaminase 1-like [Nasonia vitripennis]
comp36135_c0	1720.14	2.56	9.39	XP_003704057.1	PREDICTED: kynurenine-oxoglutarate transaminase 3-like [Megachile rotundata]
comp29610_c1	24.30	0.00	∞	XP_001607234.1	PREDICTED: kynurenine-oxoglutarate transaminase 3-like [Nasonia vitripennis]
comp22192_c0	534.83	252.92	1.08	XP_001599732.1	PREDICTED: protein disulfide-isomerase A3-like [Nasonia vitripennis]
Protease inhibitors					
comp22195_c0	11118.41	33.42	8.38	XP_003425788.1	PREDICTED: hypothetical protein LOC100677882 [Nasonia vitripennis]/region_name=“KAZAL_FS”
comp36018_c0	21799.59	571.10	5.25	XP_003424976.1	PREDICTED: hypothetical protein LOC100680056 [Nasonia vitripennis]/region_name=“KAZAL_FS”
comp44498_c8	449.23	74.76	2.59	XP_001601472.1	PREDICTED: hypothetical protein LOC100117405 [Nasonia vitripennis]/region_name=“Pacifastin_I”
comp43457_c1	214.00	105.07	1.03	XP_001602351.1	PREDICTED: hypothetical protein LOC100118367 [Nasonia vitripennis]/region_name=“SERPIN”
Recognition and binding proteins					
comp39522_c0	11298.69	38.03	8.21	NP_001155150.1	GOBP-like venom protein precursor [Nasonia vitripennis]
comp36458_c0	222.54	0.19	10.22	XP_003424242.1	PREDICTED: beta-1,3-glucan-binding protein [Nasonia vitripennis]
comp44465_c2	177.85	0.17	10.01	NP_001155040.1	low-density lipoprotein receptor-like venom protein precursor [Nasonia vitripennis]
comp39967_c0	13.21	3.29	2.00	XP_001604854.1	PREDICTED: low-density lipoprotein receptor-related protein 2-like [Nasonia vitripennis]
Others					
comp22191_c0	590.17	217.32	1.44	ACZ68113.1	calreticulin [Pteromalus puparum]
comp45101_c0	9683.63	9.36	10.01	XP_003428123.1	PREDICTED: venom allergen 3-like isoform 1 [Nasonia vitripennis]
comp40314_c0	22.65	9.07	1.32	NP_001154975.1	major royal jelly protein-like 7 precursor [Nasonia vitripennis]
comp41377_c0	4040.63	3.25	10.28	NP_001154978.1	major royal jelly protein-like 9 precursor [Nasonia vitripennis]
comp42400_c0	959.54	134.66	2.83	XP_001604366.1	PREDICTED: protein FAM151A-like [Nasonia vitripennis]
comp42334_c0	84.05	14.01	2.58	XP_003425370.1	PREDICTED: protein lethal(2)essential for life-like [Nasonia vitripennis]
comp43276_c5	596.79	0.70	9.74	CBN72521.1	venom protein A1YI24CM3 [Chelonus inanitus]
Unknown					
comp22190_c0	1507.25	3.23	8.87	NP_001155171.1	venom protein D precursor [Nasonia vitripennis]
comp45096_c0	12371.95	110.95	6.80	NP_001164344.1	venom protein G precursor [Nasonia vitripennis]
comp36121_c0	1880.93	2.15	9.77	NP_001164347.1	venom protein J precursor [Nasonia vitripennis]
comp39496_c0	3197.36	6.01	9.05	NP_001155028.1	venom protein K precursor [Nasonia vitripennis]
comp22199_c0	14424.76	30.52	8.88	NP_001155029.1	venom protein L precursor [Nasonia vitripennis]
comp39484_c0	951.21	2.35	8.66	NP_001155031.1	venom protein O precursor [Nasonia vitripennis]
comp22466_c0	2360.55	4.93	8.90	NP_001155170.1	venom protein U precursor [Nasonia vitripennis]
comp45164_c0	4856.06	5.48	9.79	NP_001155169.1	venom protein Z precursor [Nasonia vitripennis]
comp28217_c0	3818.61	4.69	9.67	XP_001601835.2	PREDICTED: hypothetical protein LOC100117668 [Nasonia vitripennis]
comp39512_c0	4969.28	8.03	9.27	XP_001603579.2	PREDICTED: hypothetical protein LOC100119874 [Nasonia vitripennis]
comp22365_c0	1440.42	2.91	8.95	XP_001605945.2	PREDICTED: hypothetical protein LOC100122343 [Nasonia vitripennis]
comp44303_c0	5418.77	4.69	10.17	XP_001606517.2	PREDICTED: hypothetical protein LOC100122910 [Nasonia vitripennis]
comp22193_c0	1795.35	13.76	7.03	XP_003426294.1	PREDICTED: hypothetical protein LOC100678001 isoform 1 [Nasonia vitripennis]
comp37024_c0	3026.09	5.75	9.04	XP_003424286.1	PREDICTED: hypothetical protein LOC100678044 [Nasonia vitripennis]
comp22198_c0	2164.89	3.08	9.46	XP_003424263.1	PREDICTED: hypothetical protein LOC100678968 [Nasonia vitripennis]
comp28774_c0	1248.40	1.84	9.41	XP_003424971.1	PREDICTED: hypothetical protein LOC100679301 [Nasonia vitripennis]
comp41010_c0	103.99	0.86	6.92	XP_003424464.1	PREDICTED: hypothetical protein LOC100679659 isoform 1 [Nasonia vitripennis]

∞indicates the infinite value from division by zero.
