# Supplementary material for: Insights into the venom composition and evolution of an endoparasitoid wasp by combining proteomic and transcriptomic analyses
Source: Sci Rep. 2016 Jan 25;6:19604. doi: 10.1038/srep19604 (PMC4726277; doi:10.1038/srep19604)
Supplement: Supplementary Information [file srep19604-s1.pdf]

Supplementary file 1 for

Insights into the venom composition and evolution of an endoparasitoid wasp by combining proteomic and transcriptomic analyses

Zhichao Yan [a, 1], Qi Fang [a, 1], Lei Wang [a], Jinding Liu [b], Yu Zhu [a], Fei Wang [a], Fei Li [a], John H. Werren [c], Gongyin Ye [a,*]

[a] *State Key Laboratory of Rice Biology & Ministry of Agriculture Key Laboratory of Agricultural Entomology, Institute of Insect Sciences, Zhejiang University, Hangzhou 310058, China*

[b] *Department of Entomology, College of Plant Protection, Nanjing Agricultural University, Nanjing 210095, China*

[c] *Department of Biology, University of Rochester, Rochester, NY 14627, USA*

* Corresponding author: Ye Gong-Yin, Institute of Insect Sciences, College of Agriculture and Biotechnology, C1152 Agro-Bio Complex, Zhejiang University, Hangzhou 310058, China (e-mail: chu@zju.edu.cn).

[1]These authors contributed equally to this work.

This file includes:

Figures S1 to S14

Tables S1, S4 and S5

Other Supporting information for this manuscript (not in this file) includes:

Tables S2 and S3

Types of venom proteins in *P. puparum*

Putative identified venom proteins were categorized into enzymes, protease inhibitors, recognition and binding proteins, others and unknown (Figure 1C). These proteins were described in more detail below:

Enzymes. Serine protease is an enzyme that cleaves peptide bonds in proteins, in which serine serves as the active amino acid.[1] Serine protease and serine protease homologues (SPHs), which have similar amino acid sequence to active serine protease but lack one of three catalytic domains, were shown to participate in several physiological processes in insects, including immune activation of Toll and prophenoloxidase (PPO) pathway.[2] They are common venom components in parasitoid wasps. Eight serine proteases and a SPH were identified in *P. puparum* venom. In *C. rubecula* venom, a SPH had been demonstrated to inhibit the host PPO activation.[3] SPH was also identified in *N. vitripennis*[4] and *A. ervi*[5] venom (Figure S9).

Metalloprotease is a protease whose catalytic mechanism involves a metal. Metalloproteases were found in some snake venoms and tick saliva, which confer antihemostatic and/or antifibrinogen specific activities.[6,7] And they are also common venom components in parasitoid wasps, which are detected in *N. vitripennis*,[4] *C. inanitus*,[8] *L. boulardi*, *L. heterotoma*,[9,10] *H. didymator*,[11] *M. hyperodae*,[12] *M. demolitor*[13] and *Eulophus pennicornis*.[14] EpMP3, a venom metalloprotease in the ectoparasitoid *E. pennicornis*, had been discovered to manipulate host development by repressing molting and metamorphosis.[14] Three metalloproteases were identified in *P. puparum* venom. Their role in *P. puparum* venom has not been demonstrated yet.

Lipase, a subclass of the esterase, is an enzyme that catalyzes the hydrolysis of lipids.[15] Lipases perform essential roles in the digestion and processing of lipids in most living organisms. It had been demonstrated that *N. vitripennis* venom could induce alterations in the host's lipid

metabolism.[16] Nine venom lipases were identified in *P. puparum*. Lipases were also identified in *N. vitripennis*[4] and *A. ervi*[17] venoms (Figure S10). These lipases in parasitoid venoms may be involved in regulation of the host's lipid metabolism.

Acid phosphatase is associated mainly with lysosomes involved in degradation. A venom acid phosphatase was previously reported in *P. puparum.*[18] In this study, three acid phosphatases were identified, and acid phosphatases were also identified in *N. vitripennis*,[4] *L. boulardi*, *L. heterotoma*[9,10] and *H. didymator*[11] venoms (Figure S11). The exact role of this enzyme in host-parasitoid interactions is still unknown, but it may be involved in the release of carbohydrates in the host, providing nutrients for the developing larvae.

A ribonuclease of the T2 family was identified in *P. puparum* venom. Ribonuclease T2 was found in the genomes of protozoans, plants, bacteria, animals and viruses with a broad range of biological roles, including scavenging of nucleic acids, degradation of self-RNA, serving as extra- or intracellular cytotoxins, and modulating host immune responses.[19] Ribonuclease T2-like protein seems to be common in *Cotesia* bracoviruses (Figure S8).[20-24] These ribonuclease T2-like proteins belong to virulence genes, which are introduced and expressed in hosts. Their roles in parasitism are still unclear. However, the convergent recruitments in both bracoviruses and parasitoid venoms suggest their potential important role in parasitism.

Inosine uridine-preferring nucleoside hydrolase catalyzes the hydrolysis of all of the commonly occurring purine and pyrimidine nucleosides into ribose and the associated base, but has a preference for inosine and uridine as substrates. This enzyme was identified in *P. puparum*, *N. vitripennis*,[4] *L. boulardi* and *L. heterotoma*[9,10] venoms (Figure S12). It was first discovered in *Crithidia fasciculate,* a parasitic protozoon which is deficient in de novo purine synthesis.[25] It was suggested that this enzyme was involved in host purine salvage for nucleic acid synthesis.

Endonuclease is an enzyme that cleaves the phosphodiester bond within a polynucleotide chain, in contrast to exonuclease, which cleaves phosphodiester bonds at the end of a polynucleotide chain. Endonucleases was identified in both *P. puparum* and *N. vitripennis* venoms.[4] They were also found in venoms of marine invertebrates[26] and snakes.[27] Their roles in venom or parasitism remain unknown.

Adenosine deaminase is another enzyme involved in purine metabolism. It is an enzyme that catalyzes the irreversible deamination of adenosine and deoxyadenosine to inosine and deoxyinosine, respectively. In *Drosophila*, adenosine deaminases cause polarization and serum-independent proliferation of imaginal disk and embryonic cells *in vitro*, by depleting of extracellular adenosine.[28] Adenosine deaminase was also identified in salivary gland of blood suck insects like the tsetse fly *Glossina*,[29] sand fly *Lutzomyia*[30] and mosquitoes.[31] Their role in parasitism is still not clear.

Glucosamine (N-acetyl)-6-sulfatase is an enzyme that catalyzes hydrolysis of the 6-sulfate groups of the N-acetyl-D-glucosamine 6-sulfate units of heparan sulfate and keratan sulfate. It is involved in the catabolism of heparin, heparan sulphate, and keratan sulphate. In *Drosophila*, an extracellular sulfatase (QSulf1) is localized on the cell surface and regulates heparan-dependent Wnt signaling through a mechanism that requires its catalytic activity.[32] Their role in parasitism is still unknown.

The protein enzyme α-amylase hydrolyses alpha bonds of large, alpha-linked polysaccharides, such as starch and glycogen, yielding glucose and maltose.[33] This enzyme in *P. puparum* venom may be involved in digestion of starch or sucrose in the host for wasp larval nutrition.

Glucose dehydrogenase is an enzyme that catalyzes D-glucose to D-glucono-1, 5-lactone.[34] This enzyme was presented in both *P. puparum* and *N. vitripennis* venoms.[4] This enzyme

participates in the pentose phosphate pathway and employs one cofactor, FAD (flavin adenine dinucleotide).

Gamma-glutamyltranspeptidas (γ-GT) is an enzyme that transfers gamma-glutamyl functional groups. This enzyme was found in *P. puparum*, *N. vitripennis*,[4] and *A. ervi*[16, 73] venoms (Figure S13). A γ-GT (Ae-γ-GT) was previously purified from *A. ervi* venom. It was suggested that this venom protein may induce apoptosis in the host ovaries by generating an alteration of the glutathione (GSH) metabolism and a consequent oxidative stress.[35] In *P. puparum*, γ-GT may have similar function with Ae-γ-GT.

Gamma-interferon-inducible lysosomal thiol reductase (GILT) is an enzyme that reduces protein disulfide bonds at low pH. In mammals, this enzyme is expressed constitutively in antigen-presenting cells and is induced by gamma-interferon in other cell types.[36] This protein plays an important role in antigen processing. And it is also implicated in the maintenance of cellular glutathione levels.[37] GILT has not been associated with venom secretions before.

Kynurenine--oxoglutarate transaminase (KAT) is an enzyme that catalyzes the transamination of kynurenine to α-keto acid. It is involved in the kynurenine pathway, which is important in both immune and neuron system.[38-40] It was first discovered as venom protein in *N. vitripennis*.[4] Three KATs were identified in *P. puparum* venom. Their role in parasitoid venom is still unknown.

Protein disulfide-isomerase (PDI) is an enzyme that catalyzes disulfide formation and isomerization.[41] PDI was found in *P. puparum*, *L. boulardi* and *L. heterotoma*[9] venoms. Like calreticulin, a common component in parasitoid venoms, PDI is another chaperone mainly present in endoplasmic reticulum and involved in protein refolding.

Protease inhibitors. Uncontrolled enzymes that break down proteins are potentially very

damaging in living systems, so their activities need to be strictly regulated. The suppression of the proteases with inhibitors is one of the important mechanisms for controlling of excessive activity.[42] Four protease inhibitors were identified in *P. puparum* venom. They were a twin serpin (Figure S14), a protease inhibitor with similarity to pacifastin and two Kazal type protease inhibitors, respectively. Serpin is a serine protease inhibitor involved in a lot of physiological process, including immune activation of both Toll and PPO pathway.[43-45] A serpin (LbSPNy) in *L. boulardi* venom was demonstrated to suppress host melanization by inhibiting the activation of PPO pathway.[46] Pacifastin, an inhibitor of the PPO-activating enzyme in *Pacifastacus leniusculus*, also negatively regulates the PPO activation.[47,48] These inhibitors present in *P. puparum* venom may be involved in self-protection from the venom toxicity or suppression of the host physiological process.

Recognition and binding proteins. β-1, 3-glucan-binding protein (βGRP) was identified in both *P. puparum* and *N. vitripennis* venoms.[4] Pattern recognition molecules serve as biosensors in the activation of innate immune responses in both vertebrates and invertebrates. The family of βGRPs is involved in the recognition of β-1, 3-glucans of fungi and Gram-negative bacteria. βGRPs from several arthropods have been implicated in the activation of a protease cascade that leads to PPO activation[49] and induction of antimicrobial peptide genes.[50] This protein in parasitoid venom may be involved in host's immunosuppression.

Low-density lipoprotein (LDL) receptor plays a central role in mammalian cholesterol metabolism. It binds and transports LDL into cells by endocytosis. LDL receptor-related proteins were identified in both *P. puparum* and *N. vitripennis*[4] venoms. An insect homolog of the vertebrate very low density lipoprotein receptor was found to mediate this endocytosis of lipophorins.[51] In host- parasitoid interaction, it was proposed that this protein could possibly be

responsible for the internalization of lipophorins in the fat body.[51,52]

Odorant-binding protein (OBP) is a small secreted protein that is important in olfactory communication in insects.[53] This protein participates in solubilization and transport of small hydrophobic odorant molecules and pheromones. The general odorant-binding venom protein in *P. puparum* (PpOBP) was previously identified and characterized.[54] *PpOBP* mRNA was specifically expressed in venom gland. And the mRNA level was significantly up-regulated by feeding and parasitism. OBPs were also found in *N. vitripennis*[4] and *C. inanitus*[8] venoms. Their role in venom or parasitism is not described yet. It was suggested that they could be associated with host selection or oviposition behavior.[55]

Others. Calreticulin is a multifunctional Ca^{2+} binding protein and a common component of parasitoid venom. This protein was previously identified in *P. puparum,*[56] *N. vitripennis,*[4] *C. rubecula,*[57] *H. didymator,*[11] *L. boulardi, L. heterotoma*[9,10] and *M. hyperodae*[12] venoms (Figure S7). A protein similar to calreticulin was discovered in *C. rubecula* venom to inhibit encapsulation of host hemocytes.[57] It was suggested that venom calreticulin could compete for binding sites with host hemocyte calreticulin, which was known to mediate early hemocyte encapsulation reactions.[58] *P. puparum* calreticulin (PpCRT) was also demonstrated to conduct the similar function by suppressing the expression of encapsulation related genes.[59] In *N. vitripennis*, calreticulin knowdown by RNAi in the wasp resulted in increased melanization of envenomated hosts.[60]

Two venom proteins that showed sequence similarities with venom antigen 5 were found in *P. puparum* venom. Venom antigen 5 was also identified in venoms from *P. puparum, N. vitripennis*,[4] *M. hyperodae*[12] and *C. inanitus*[8] (Figure S6). Venom antigen 5 is the major allergy-inducing toxin in venoms of social Hymenoptera.[61] It was commonly identified in venoms from

Vespoidea (Figure S6). It seems to be an ancient venom protein in Hymenoptera with a conserved but still unknown function.

Major royal jelly protein (MRJP) plays an important role in caste determination of honey bee.[62] This protein regulates the larvae development and induces queen differentiation. MRJP was also previously investigated in honeybee venom.[63,64] Two MRJP-like proteins were identified in *P. puparum* venom. And MRJPs were demonstrated to be present in *Nasonia* genome,[65] which contained the largest currently-known MRJP gene family, suggesting that MRJPs were important both in caste-dependent and -independent insects. However, their exact role in parasitism is still unknown.

References

1. Page, M. J. & Di Cera, E. Serine peptidases: classification, structure and function. *Cell. Mol. Life Sci.* **65**, 1220-1236 (2008).
2. Jang, I. H., Nam, H. J. & Lee, W. J. CLIP-domain serine proteases in *Drosophila* innate immunity. *BMB Rep.* **41**, 102-107 (2008).
3. Asgari, S., Zhang, G., Zareie, R. & Schmidt, O. A serine proteinase homolog venom protein from an endoparasitoid wasp inhibits melanization of the host hemolymph. *Insect Biochem. Mol. Biol.* **33**, 1017-1024 (2003).
4. de Graaf, D. C. *et al.* Insights into the venom composition of the ectoparasitoid wasp *Nasonia vitripennis* from bioinformatic and proteomic studies. *Insect Mol. Biol.* **19**, 11-26 (2010).
5. Colinet, D. *et al.* Identification of the main venom protein components of *Aphidius ervi*, a parasitoid wasp of the aphid model *Acyrthosiphon pisum*. *BMC Genomics* **15**, 342 (2014).
6. Jia, L. G., Shimokawa, K. I., Bjarnason, J. B. & Fox, J. W. Snake venom metalloproteinases: structure, function and relationship to the Adams family of proteins. *Toxicon* **34**, 1269-1276 (1996).
7. Francischetti, I. M. B., Mather, T. N. & Ribeiro, J. M. C. Cloning of a salivary gland metalloprotease and characterization of gelatinase and fibrin(ogen)lytic activities in the saliva of the Lyme disease tick vector *Ixodes scapularis*. *Biochem. Biophys. Res. Commun.* **305**, 869-875 (2003).
8. Vincent, B. *et al.* The venom composition of the parasitic wasp *Chelonus inanitus* resolved by combined expressed sequence tags analysis and proteomic approach. *BMC Genomics* **11**, 693 (2010).
9. Goecks, J. *et al.* Integrative approach reveals composition of endoparasitoid wasp venoms. *Plos One* **8** (2013).
10. Colinet, D. *et al.* Extensive inter- and intraspecific venom variation in closely related parasites targeting the same host: the case of *Leptopilina* parasitoids of *Drosophila*. *Insect Biochem. Mol. Biol.* **43**, 601-611 (2013).
11. Doremus, T. *et al.* Venom gland extract is not required for successful parasitism in the polydnavirus-associated endoparasitoid *Hyposoter didymator* (Hym. Ichneumonidae) despite the presence of numerous novel and conserved venom proteins. *Insect Biochem. Mol. Biol.* **43**, 292-307 (2013).
12. Crawford, A. M. *et al.* The constituents of *Microctonus sp.* parasitoid venoms. *Insect Mol. Biol.* **17**, 313-324 (2008).
13. Burke, G. R. & Strand, M. R. Systematic analysis of a wasp parasitism arsenal. *Mol. Ecol.* **23**, 890-901 (2014).
14. Price, D. R. G. *et al.* A venom metalloproteinase from the parasitic wasp *Eulophus pennicornis* is toxic towards its host, tomato moth (*Lacanobia oleracae*). *Insect Mol. Biol.* **18**, 195-202 (2009).
15. Hasan, F., Shah, A. A. & Hameed, A. Methods for detection and characterization of lipases: A comprehensive review. *Biotechnol. Adv.* **27**, 782-798 (2009).
16. Rivers, D. B. & Denlinger, D. L. Venom-induced alterations in fly lipid metabolism and its impact on larval

development of the ectoparasitoid *Nasonia vitripennis* (Walker) (Hymenoptera: Pteromalidae). *J. Invertebr. Pathol.* **66**, 104-110 (1995).
17. Nguyen, T. T. A. *et al.* Early presence of an enolase in the oviposition injecta of the aphid parasitoid *Aphidius ervi* analyzed with chitosan beads as artificial hosts. *J. Insect Physiol.* **59**, 11-18 (2013).
18. Zhu, J. Y., Ye, G. Y. & Hu, C. Molecular cloning and characterization of acid phosphatase in venom of the endoparasitoid wasp *Pteromalus puparum* (Hymenoptera: Pteromalidae). *Toxicon* **51**, 1391-1399 (2008).
19. Luhtala, N. & Parker, R. T2 family ribonucleases: ancient enzymes with diverse roles. *Trends Biochem. Sci.* **35**, 253-259 (2010).
20. Jancek, S. *et al.* Adaptive selection on Bracovirus genomes drives the specialization of *Cotesia* parasitoid wasps. *Plos One* **8**, 11 (2013).
21. Espagne, E. *et al.* Genome sequence of a polydnavirus: insights into symbiotic virus evolution. *Science* **306**, 286-289 (2004).
22. Choi, J. Y. *et al.* Sequence and gene organization of 24 circles from the *Cotesia plutellae* bracovirus genome. *Arch. Virol.* **154**, 1313-1327 (2009).
23. Chen, Y. F. *et al.* Deep sequencing of Cotesia vestalis bracovirus reveals the complexity of a polydnavirus genome. *Virology* **414**, 42-50 (2011).
24. Bezier, A. *et al.* Functional endogenous viral elements in the genome of the parasitoid wasp *Cotesia congregata*: insights into the evolutionary dynamics of bracoviruses. *Philos. Trans. R. Soc. Lond. B. Biol. Sci.* **368** (2013).
25. Gopaul, D. N., Meyer, S. L., Degano, M., Sacchettini, J. C. & Schramm, V. L. Inosine-uridine nucleoside hydrolase from Crithidia fasciculata. Genetic characterization, crystallization, and identification of histidine 241 as a catalytic site residue. *Biochemistry* **35**, 5963-5970 (1996).
26. Neeman, I., Calton, G. J. & Burnett, J. W. Purification of an endonuclease present in *Chrysaora quinquecirrha* venom. *Proc. Soc. Exp. Biol. Med.* **166**, 374-382 (1981).
27. Dhananjaya, B. L. & D'Souza, C. J. M. An overview on nucleases (DNase, RNase, and phosphodiesterase) in snake venoms. *Biochemistry (Mosc.)* **75**, 1-6 (2010).
28. Zurovec, M., Dolezal, T., Gazi, M., Pavlova, E. & Bryant, P. J. Adenosine deaminase-related growth factors stimulate cell proliferation in *Drosophila* by depleting extracellular adenosine. *Proc. Natl. Acad. Sci. U. S. A.* **99**, 4403-4408 (2002).
29. Li, S., Kwon, J. & Aksoy, S. Characterization of genes expressed in the salivary glands of the tsetse fly, *Glossina morsitans morsitans*. *Insect Mol. Biol.* **10**, 69-76 (2001).
30. Valenzuela, J. G., Garfield, M., Rowton, E. D. & Pham, V. M. Identification of the most abundant secreted proteins from the salivary glands of the sand fly *Lutzomyia longipalpis*, vector of *Leishmania chagasi*. *J. Exp. Biol.* **207**, 3717-3729 (2004).
31. Ribeiro, J. M. C., Charlab, R. & Valenzuela, J. G. The salivary adenosine deaminase activity of the mosquitoes *Culex quinquefasciatus* and *Aedes aegypti*. *J. Exp. Biol.* **204**, 2001-2010 (2001).
32. Dhoot, G. K. *et al.* Regulation of Wnt signaling and embryo patterning by an extracellular sulfatase. *Science* **293**, 1663-1666 (2001).
33. Prakash, O. & Jaiswal, N. alpha-Amylase: an ideal representative of thermostable enzymes. *Appl. Biochem. Biotechnol.* **160**, 2401-2414 (2010).
34. Ferri, S., Kojima, K. & Sode, K. Review of glucose oxidases and glucose dehydrogenases: a bird's eye view of glucose sensing enzymes. *J. Diabetes Sci. Technol.* **5**, 1068-1076 (2011).
35. Falabella, P. *et al.* A γ-glutamyl transpeptidase of *Aphidius ervi* venom induces apoptosis in the ovaries of host aphids. *Insect Biochem. Mol. Biol.* **37**, 453-465 (2007).
36. West, L. C. & Cresswell, P. Expanding roles for GILT in immunity. *Curr. Opin. Immunol.* **25**, 103-108 (2013).
37. Chiang, H. S. & Maric, M. Lysosomal thiol reductase negatively regulates autophagy by altering glutathione synthesis and oxidation. *Free Radical Biol. Med.* **51**, 688-699 (2011).
38. Alban, B. *et al.* Aryl hydrocarbon receptor control of a disease tolerance defence pathway. *Nature* **511**, 184-190 (2014).
39. Mándi, Y. & Vécsei, L. The kynurenine system and immunoregulation. *J. Neural Transm.* **119**, 197-209 (2012).
40. Vecsei, L., Szalardy, L., Fulop, F. & Toldi, J. Kynurenines in the CNS: recent advances and new questions. *Nat. Rev. Drug Discov.* **12**, 64-82 (2013).
41. Gruber, C. W., Cemazar, M., Heras, B., Martin, J. L. & Craik, D. J. Protein disulfide isomerase: the structure of oxidative folding. *Trends Biochem. Sci.* **31**, 455-464 (2006).

42. Rawlings, N. D., Tolle, D. P. & Barrett, A. J. Evolutionary families of peptidase inhibitors. *Biochem. J.* **378**, 705-716 (2004).
43. Polanowski, A. & Wilusz, T. Serine proteinase inhibitors from insect hemolymph. *Acta Biochim. Pol.* **43**, 445-453 (1996).
44. Gubb, D., Sanz-Parra, A., Barcena, L., Troxler, L. & Fullaondo, A. Protease inhibitors and proteolytic signalling cascades in insects. *Biochimie* **92**, 1749-1759 (2010).
45. Jiang, R. *et al.* 93-kDa twin-domain serine protease inhibitor (Serpin) has a regulatory function on the beetle Toll proteolytic signaling cascade. *J. Biol. Chem.* **286**, 35087-35095 (2011).
46. Colinet, D. *et al.* A serpin from the parasitoid wasp *Leptopilina boulardi* targets the *Drosophila* phenoloxidase cascade. *Dev. Comp. Immunol.* **33**, 681-689 (2009).
47. Liang, Z., Sottrup-Jensen, L., Aspan, A., Hall, M. & Soderhall, K. Pacifastin, a novel 155-kDa heterodimeric proteinase inhibitor containing a unique transferrin chain. *Proc. Natl. Acad. Sci. U. S. A.* **94**, 6682-6687 (1997).
48. Liu, H. *et al.* Phenoloxidase is an important component of the defense against Aeromonas hydrophila Infection in a crustacean, *Pacifastacus leniusculus*. *J. Biol. Chem.* **282**, 33593-33598 (2007).
49. Bilej, M. *et al.* Distinct carbohydrate recognition domains of an invertebrate defense molecule recognize Gram-negative and Gram-positive bacteria. *J. Biol. Chem.* **276**, 45840-45847 (2001).
50. Kim, Y. S. *et al.* Gram-negative bacteria-binding protein, a pattern recognition receptor for lipopolysaccharide and beta-1,3-glucan that mediates the signaling for the induction of innate immune genes in *Drosophila melanogaster* cells. *J. Biol. Chem.* **275**, 32721-32727 (2000).
51. Dantuma, N. P. *et al.* An insect homolog of the vertebrate very low density lipoprotein receptor mediates endocytosis of lipophorins. *J. Lipid Res.* **40**, 973-978 (1999).
52. Danneels, E. L., Rivers, D. B. & de Graaf, D. C. Venom proteins of the parasitoid wasp *Nasonia vitripennis*: recent discovery of an untapped pharmacopee. *Toxins* **2**, 494-516 (2010).
53. Ha, T. S. & Smith, D. P. Odorant and pheromone receptors in insects. *Front. Cell. Neurosci.* **3** (2009).
54. Wang, L., Zhu, J. Y., Qian, C., Fang, Q. & Ye, G. Y. Venom of the parasitoid wasp *Pteromalus puparum* contains an odorant binding protein. *Arch. Insect Biochem. Physiol.* **88**, 101-110 (2015).
55. Heavner, M. E. *et al.* Partial venom gland transcriptome of a *Drosophila* parasitoid wasp, *Leptopilina heterotoma*, reveals novel and shared bioactive profiles with stinging Hymenoptera. *Gene* **526**, 195-204 (2013).
56. Zhu, J. Y., Fang, Q., Wang, L., Hu, C. & Ye, G. Y. Proteomic analysis of the venom from the endoparasitoid wasp *Pteromalus puparum* (Hymenoptera: Pteromalidae). *Arch. Insect Biochem. Physiol.* **75**, 28-44 (2010).
57. Zhang, G., Schmidt, O. & Asgari, S. A calreticulin-like protein from endoparasitoid venom fluid is involved in host hemocyte inactivation. *Dev. Comp. Immunol.* **30**, 756-764 (2006).
58. Wang, L. *et al.* Molecular cloning and functional study of calreticulin from a lepidopteran pest, *Pieris rapae*. *Dev. Comp. Immunol.* **38**, 55-65 (2012).
59. Wang, L. *et al.* Inhibition of host cell encapsulation through inhibiting immune gene expression by the parasitic wasp venom calreticulin. *Insect Biochem. Mol. Biol.* **43**, 936-946 (2013).
60. Siebert, A. L., Wheeler, D. & Werren, J. H. A new approach for investigating venom function applied to venom calreticulin in a parasitoid wasp. *Toxicon* **(in process)** (2015).
61. Lu, G., Villalba, M., Coscia, M. R., Hoffman, D. R. & King, T. P. Sequence analysis and antigenic cross-reactivity of a venom allergen, antigen 5, from hornets, wasps, and yellow jackets. *J. Immunol.* **150**, 2823-2830 (1993).
62. Kamakura, M. Royalactin induces queen differentiation in honeybees. *Nature* **473**, 478-483 (2011).
63. Peiren, N. *et al.* The protein composition of honeybee venom reconsidered by a proteomic approach. *Biochim. Biophys. Acta* **1752**, 1-5 (2005).
64. Peiren, N. *et al.* Proteomic analysis of the honey bee worker venom gland focusing on the mechanisms of protection against tissue damage. *Toxicon* **52**, 72-83 (2008).
65. Werren, J. H. *et al.* Functional and evolutionary insights from the genomes of three parasitoid *Nasonia* species. *Science* **327**, 343-348 (2010).

Table S1. Comparison of two methods used for signal peptide analysis

Method 1	Y	N	Y	N	Identity between two methods
Method 2	Y	N	N	Y	
Number of transcripts	1281	10164	79	39	99.00%

Method 1: signal peptide analysis using retrieved reference sequences; Method 2: signal peptide analysis using translated proteins; Y: identified with signal peptides by the methods; N: identified without signal peptides by the methods.

Table S4. Number of *P. puparum* venom protein hits using different cutoffs

**Bitscore cutoff**	**0**	**20**	**30**	**40**	**50**	**60**	**70**	**80**	**90**	**100**
Both	21	21	21	20	18	16	14	13	12	12
No hit	11	11	11	11	14	17	21	23	25	25
NvVen only	24	24	24	25	25	24	22	21	21	21
OEPVen only	14	14	14	14	13	13	13	13	12	12

The cutoffs used are combinations of e-value >= 1e-5 and bit score greater than or equal to bit score cutoffs.

Table S5. Primers used for Real-time PCR verification of putative venom proteins from *Pteromalus puparum*

	Sequence ID	Forward Primer	Reverse Primer
1	comp45178_c0	GCTGAACTTTTCGAGGATGC	AGCGTCTTGGCCAATTTTT
2	comp39547_c0	CTCACAAGCCGCACATTAGA	TGTGAATCGCAAAGAACGAG
3	comp29468_c0	CGCAGCCTTTCATTTTCAAT	TAGAGGTCCACCCGAATCAC
4	comp39512_c0	CGTCTGGCTAAAGAAAACGG	ATTTTGTCCCACGTCTTTGC
5	comp22199_c0	ATGCTATTCCCACTGCCATC	TGCTGGTCTATTCCCGTTTC
6	comp43143_c1	ATTCTTACGGCTGGTCCCTT	ATCCAAAACCAGCGATTGTC
7	comp36032_c0	GGCATACGCCATTTTTCCTA	TCGTGAAGCTCGACAAACAC
8	comp39522_c0	AAGCCCGATTCAGGTAAC	CCGAGTCCAAAATACACG
9	comp28596_c0	AAGTGATTCTCCGTCACGCT	GGTGTGCCAAGGCTTAATGT
10	comp22365_c0	CCGTTCAATTTGGCTTTAGG	GCAGCTTTCATACCAGCCTC
11	comp36135_c0	TGGAGATTGGGATGGGTTTA	AGTTCATCCTCGAACGCAAT
12	comp44819_c3	AGTGATGACACTCGCACTGG	TATGGCGCACCAGAAGTGTA
13	comp36103_c0	GCTAATCCAAAGCGGAGACA	CACATCAGGTAGTTGCTCGC
14	comp22216_c0	GTCCAGAATGCGAATCTCGT	GTGCATTTTCCGTTGACCTT
15	comp44303_c0	AATGGCGCAGTTCAAATACC	GAGAAATTCATCGGAACGGA
16	comp28217_c0	ATGGCAACCTGTAACGGAAG	TGTGCCGTGATTTTAAGACG
17	comp36121_c0	GGTCGTGATCTCGGAAAAGA	GGACCAGCAGTCACCAGATT
18	comp45164_c0	GAAAAGGTAGCAGCCTGGTG	TGCTGTTTGGCTTTGTATGC
19	comp41377_c0	CGGTAGCTGAATTCCAGGAG	ATCGCAAATTGCCTCGTTAC
20	comp42418_c0	GGTAATTGGGCTCGCATAGA	CCTCTGTTGCTGAGAGGGTC
21	comp45101_c0	TCAAGCACTATTGCAGTGCC	ATGGCAGGCAAATCTGAATC
22	comp44465_c2	AGTCAGCATCCATCTGTCCC	GCGGAAACCTTCTGACACAT
23	comp42555_c0	GCCCTGTTTCTTAAGCGTTG	ATGCATTAATTCCGTTTCCG
24	comp22275_c0	TTGTCAAGATGACTCCACGG	CGAATCCATGCTTCTTCGTT
25	comp29111_c0	GCAAATGACCCAGAAGGAAA	ATGTCTCGTTCGAAGCTCGT
26	comp36458_c0	GCCTCCGTATTTCCATACCA	ATTCTTCGGCTTCACGATGT
27	comp28774_c0	AGTATCGCCGTAAATGGTGC	CCAATCGATTCCTCCACTGT
28	comp28533_c0	ACTTCTACGTCATGGGTGGC	GTCCTTCGTGATCGGAGTGT
29	comp36060_c0	TTTATTGCAGCCAGCATGAG	CTTTTGGAAGCTTCAATGCC
30	comp45096_c0	GGTCACACAGGTGTGCTT	AGTAACCGCCGCTAGATT
31	comp39496_c0	TTAAAGTGTCCGACGAGCA	GAGGGCCTTCGCATAAGTA
32	comp22466_c0	TCCGGATGTGCATAAAATG	TCCTTTTTCGTGGTGTCAA
33	comp22190_c0	CACGGACAGGAACACGTTA	TGGGTCTATTTCAGCACGA
34	comp22195_c0	GGCATTTGGATGTGAAGAAG	TCGGCCTTATAATCTTCAGC
reference	18S rRNA	CGAGCGATGAACCGACAG	CGGGGAGGTAGTGACGAA

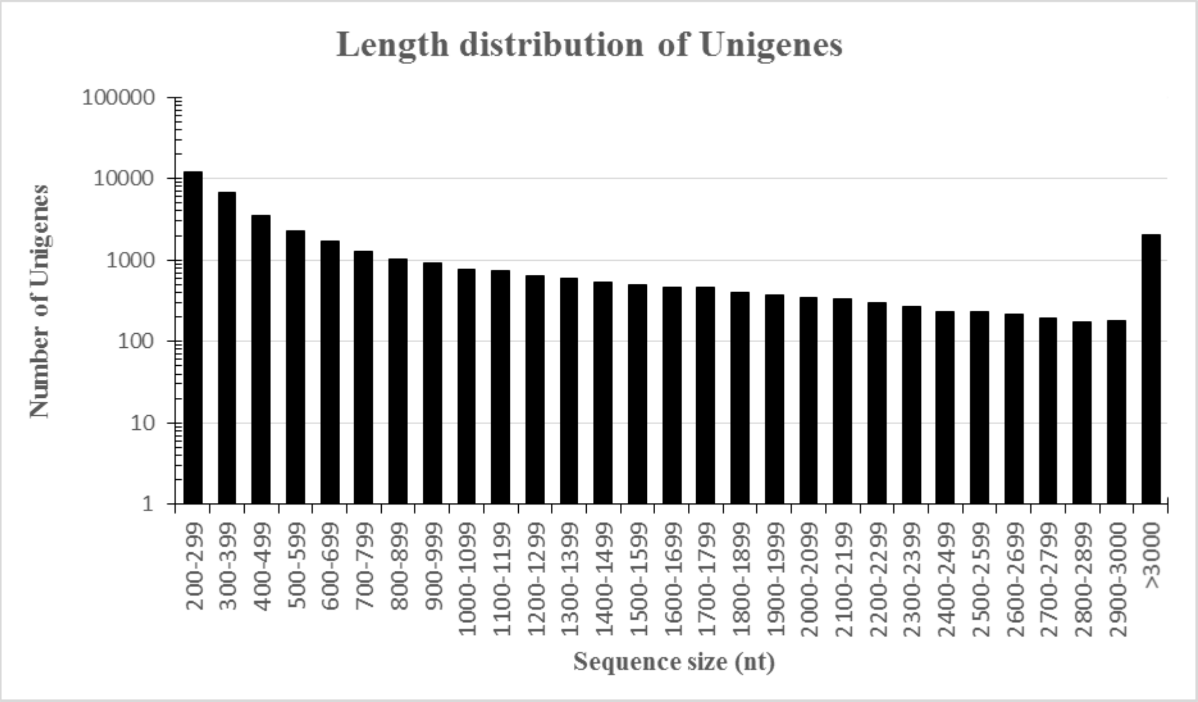

Figure S1. Distribution of lengths of the unigenes from *P. puparum* transcriptome.

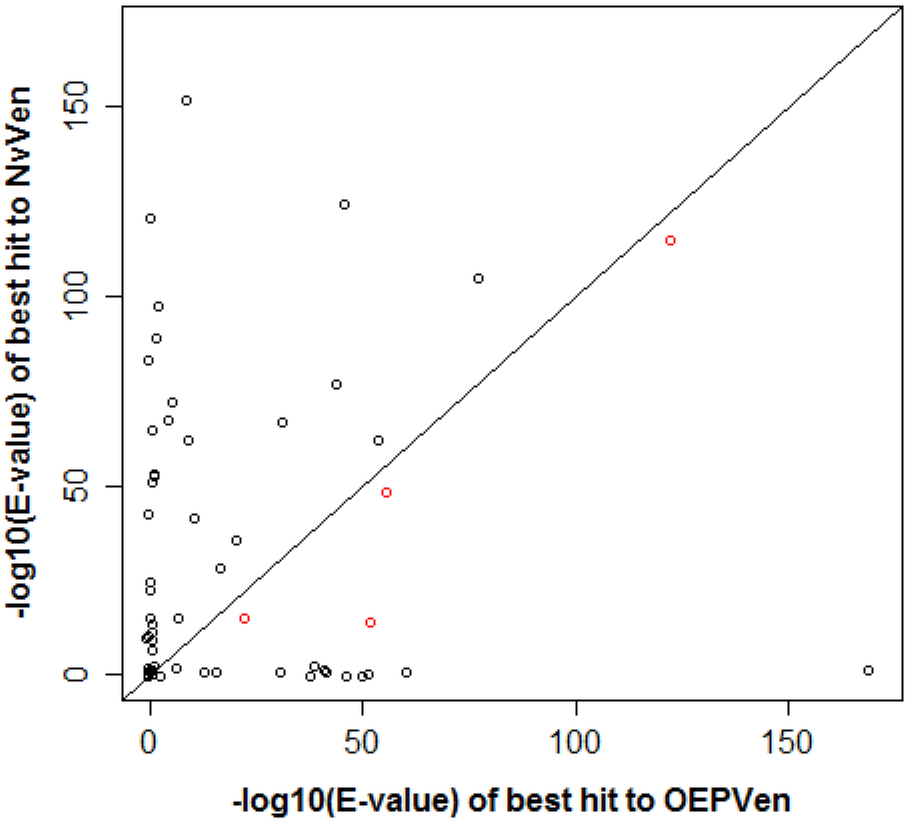

Figure S2. Distribution of –log10(E-value) of *P. puparum* venom proteins best hit in *N. vitripennis* and other endoparasitoid venoms. Red circles indicate venom proteins of *P. puparum* which hit (e-value ≤ 1e^{-5} and bit score ≥ 50) both to venom proteins from *N. vitripennis* and other endoparasitoid and show higher similarities to venom proteins other endoparasitoids from than *N. vitripennis*. *Nv*Ven: venom proteins from *N. vitripennis*. OEPVen: venom proteins from other endoparasitoid wasps.

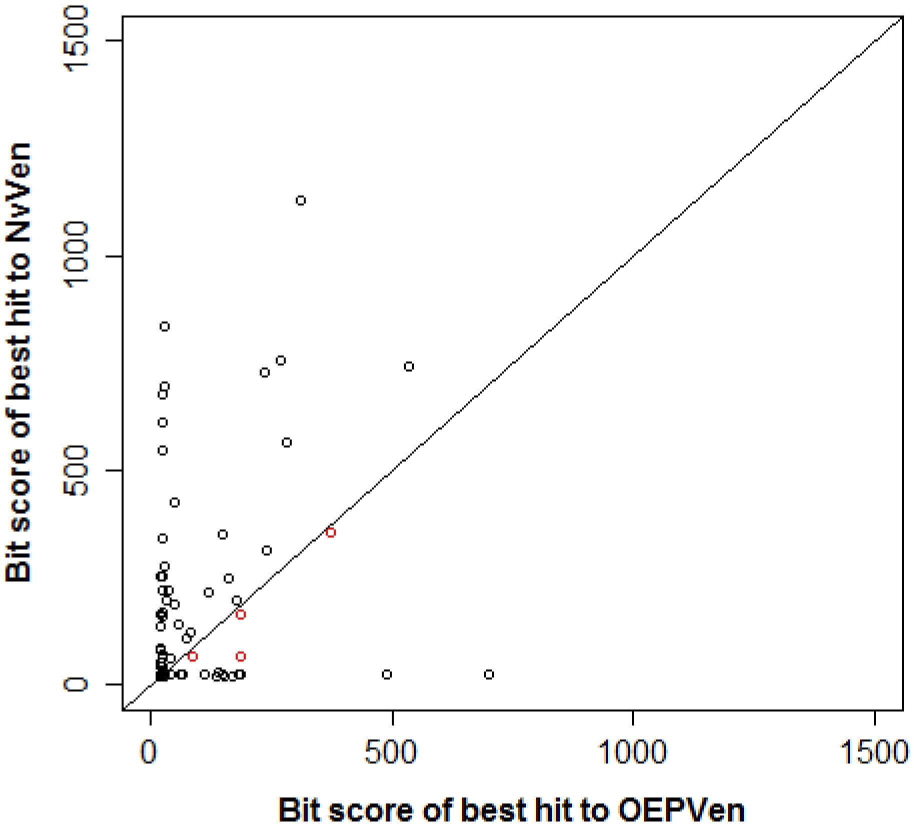

Figure S3. Distribution of bit score of *P. puparum* venom proteins best hit in *N. vitripennis* and other endoparasitoid venoms. Red circles indicate venom proteins of *P. puparum* which hit (e-value ≤ 1e^{-5} and bit score ≥ 50) both to venom proteins from *N. vitripennis* and other endoparasitoid and show higher similarities to venom proteins other endoparasitoids from than *N. vitripennis*. *Nv*Ven: venom proteins from *N. vitripennis*. OEPVen: venom proteins from other endoparasitoid wasps.

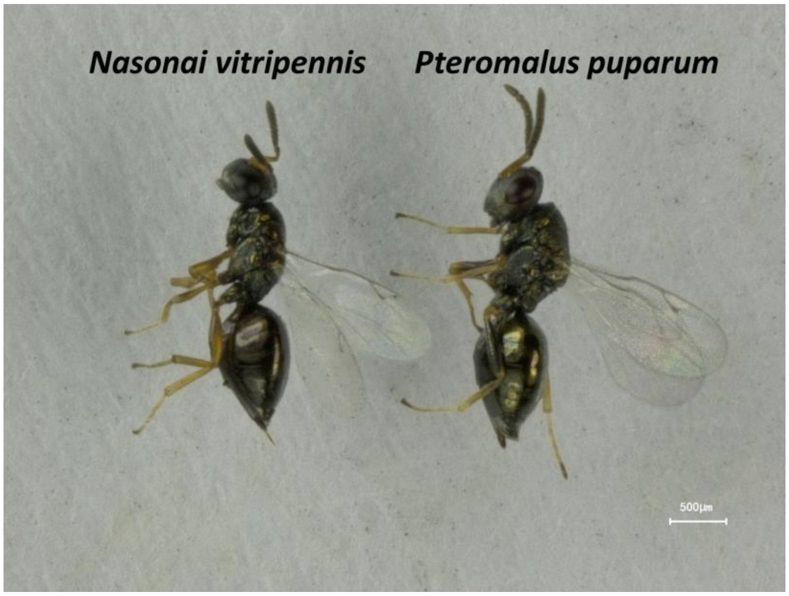

Figure S4. Morphological comparison of *Nasonia vitripennis* and *Pteromalus puparum* female adults.

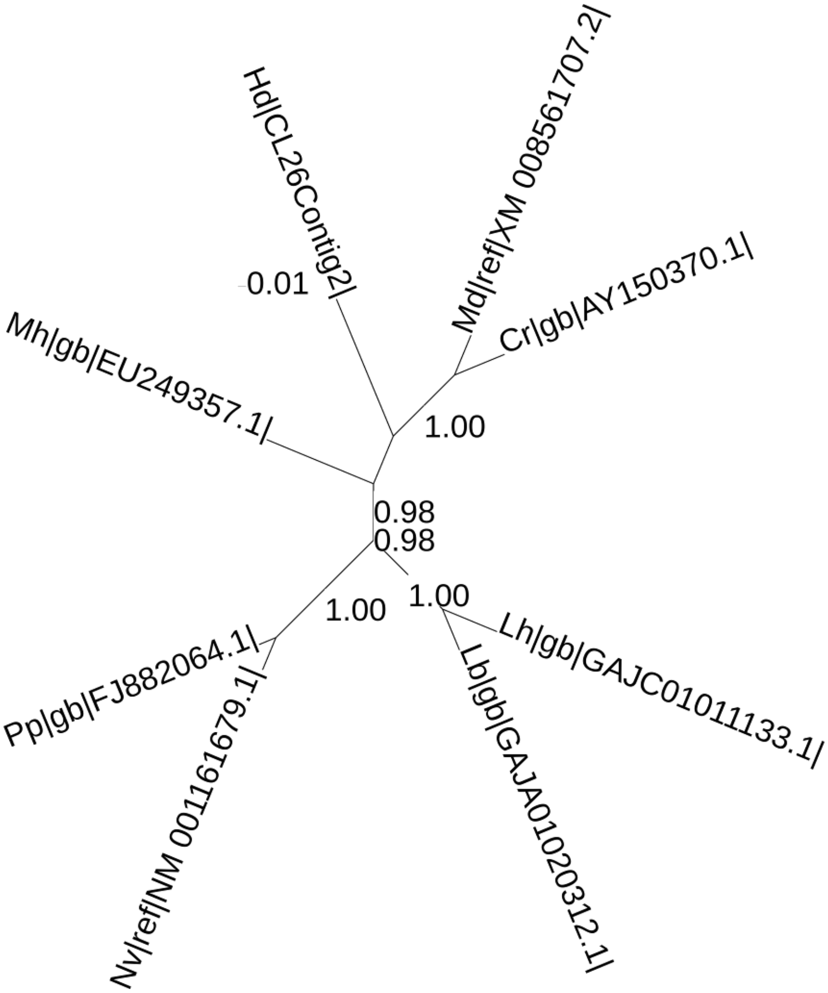

Figure S5. Unrooted phylogenetic tree of calreticulin coding sequences from parasitoid wasps. Pp: *P. puparum*; Nv: *N. vitripennis*; Cr: *Cotesia rubecula*; Hd: *Hyposoter didymator*; Lb: *Leptopilina boulardi*; Lh: *L. heterotoma*; Mh: *Microctonus hyperodae*; Md: *Microplitis demolitor*.

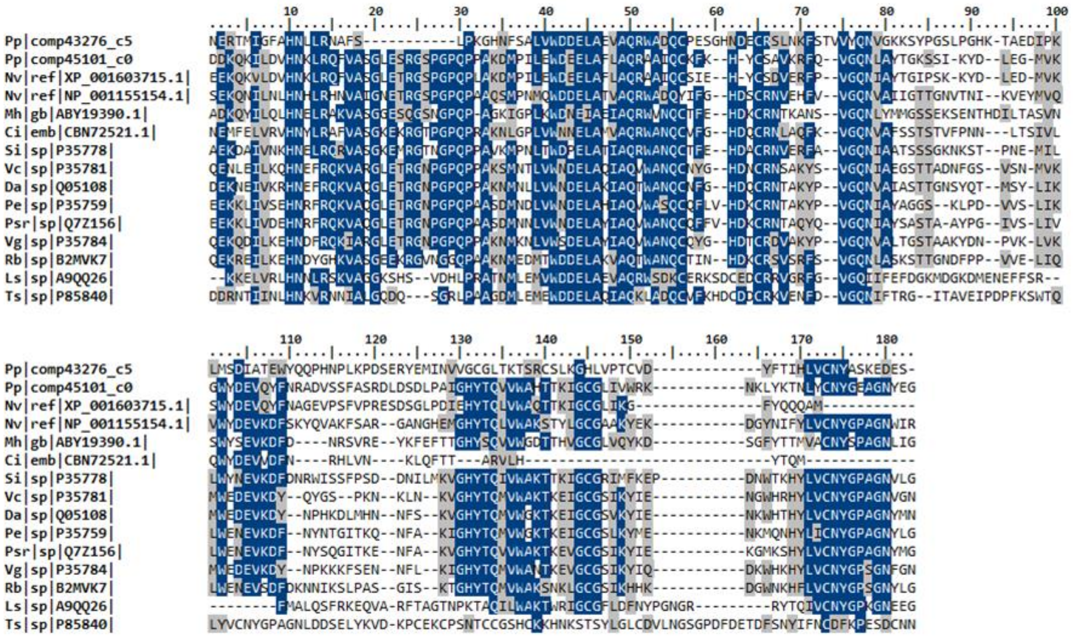

Figure S6. Sequences alignment of "SCP" domain in venom allergen 5-like venom proteins from arthropods. Residues identical or similar are highlighted in black and grey, respectively. Pp: *P. puparum*; Nv: *N. vitripennis*; Mh: *M. hyperodae*; Ci: *Chelonus inanitus*; Si: *Solenopsis invicta*; Vc: *Vespa crabro*; Da: *Dolichovespula arenaria*; Pe: *Polistes exclamans*; Psr: *Polybia scutellaris rioplatensis*; Vg: *Vespula germanica*; Rb: *Rhynchium*

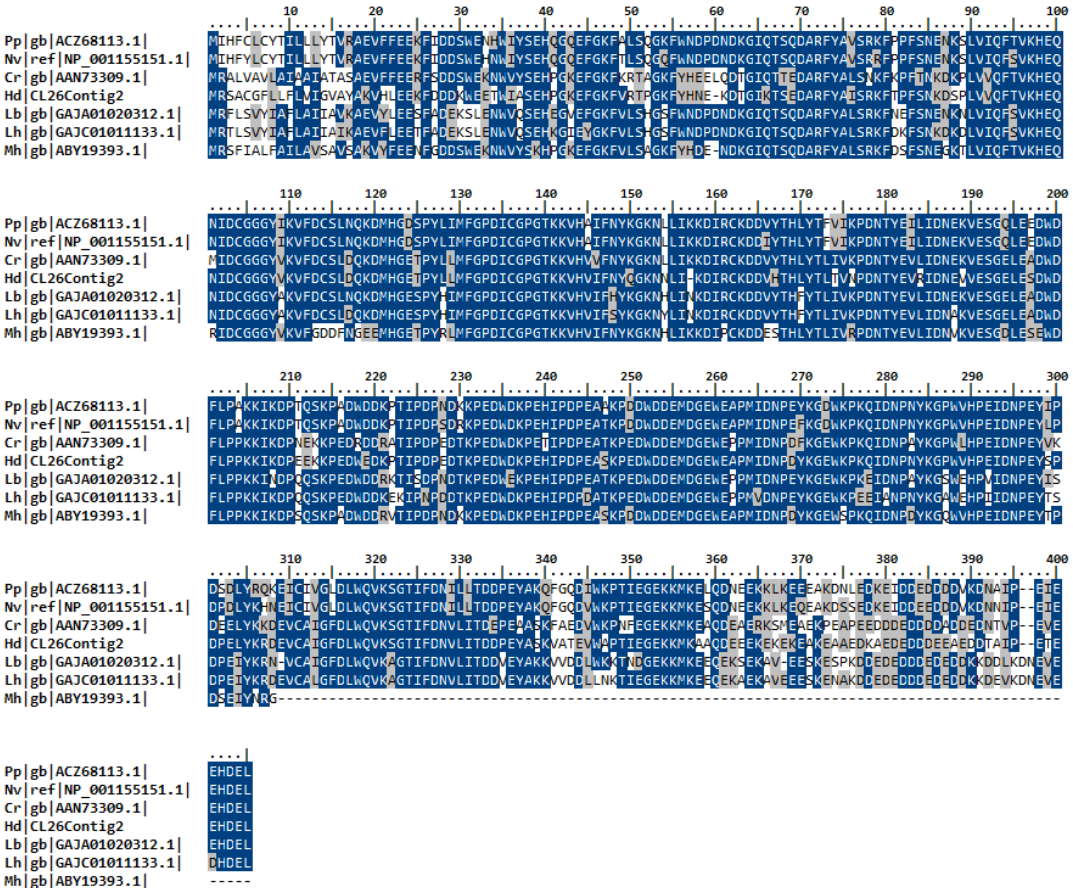

Figure S7. Sequence alignment of calreticulin from parasitoid wasp venoms. Residues identical or similar are highlighted in black and grey, respectively. Pp: *P. puparum*; Nv: *N. vitripennis*; Cr: *Cotesia rubecula*; Hd: *Hyposoter didymator*; Lb: *Leptopilina boulardi*; Lh: *L. heterotoma*; Mh: *M. hyperodae*.

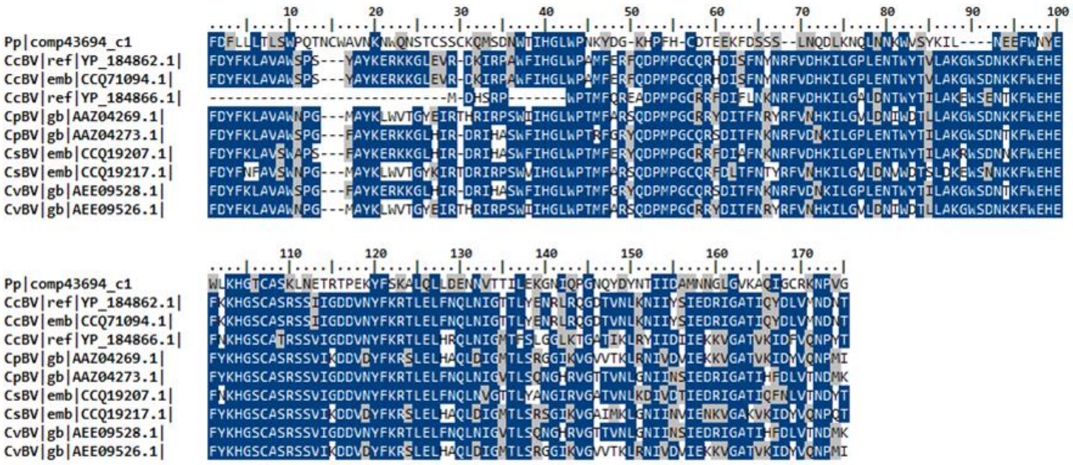

Figure S8. Sequences alignment of "Ribonuclease_T2" domain from *P. puparum* venom and PDVs. Residues identical or similar are highlighted in black and grey, respectively. Pp: *P. puparum*; CcBV: *Cotesia congregata* bracovirus; CpBV: *Cotesia plutellae* bracovirus; CsBV: *Cotesia sesamiae* Kitale bracovirus; CvBV: *Cotesia vestalis* bracovirus.

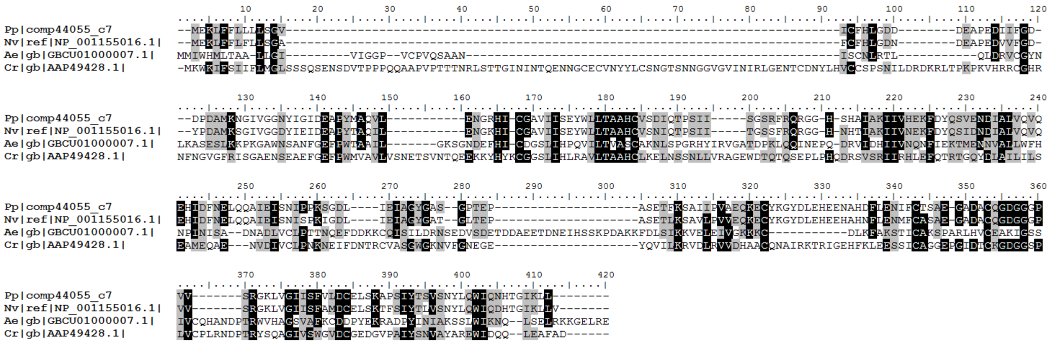

Figure S9. Sequence alignment of serine proteinase homolog from parasitoid wasp venoms. Pp: *P. puparum*; Nv: *N. vitripennis*; Ae: *A. ervi*; Cr: *C. rubecula*.

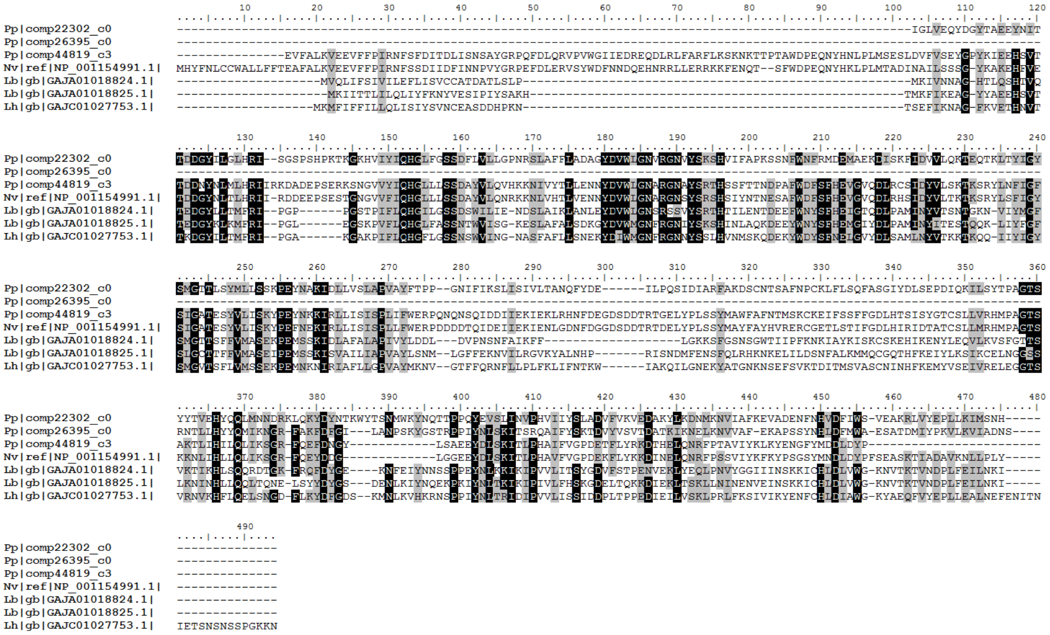

Figure S10. Sequence alignment of lipase-like proteins from parasitoid wasp venoms. Pp: *P. puparum*; Nv: *N. vitripennis*; Lb: *L. boulardi*; Lh: *L. heterotoma*.

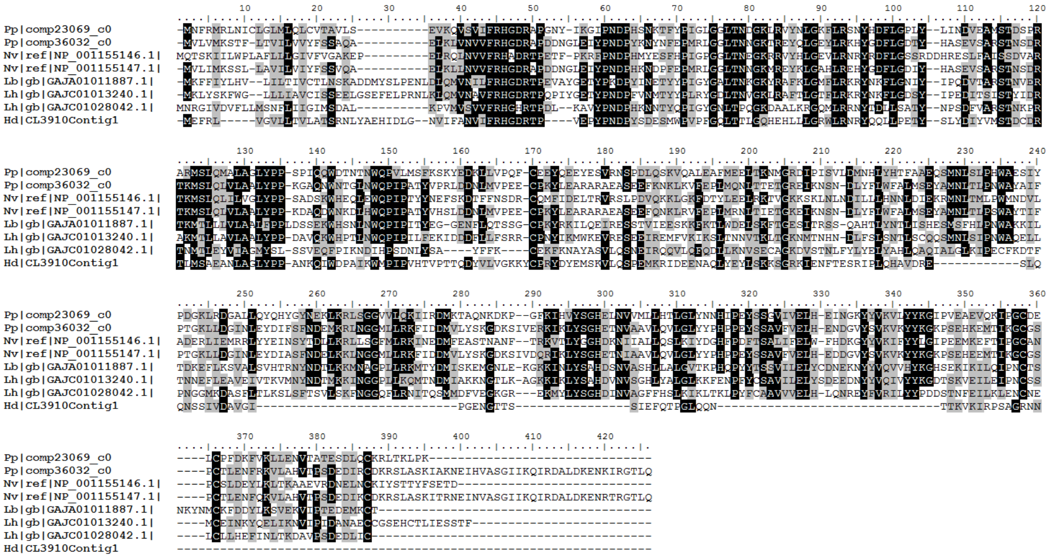

Figure S11. Sequence alignment of acid phosphatase from parasitoid wasp venoms. Pp: *P. puparum*; Nv: *N. vitripennis*; Lb: *L. boulardi*; Lh: *L. heterotoma*; Hd: *H. didymator*.

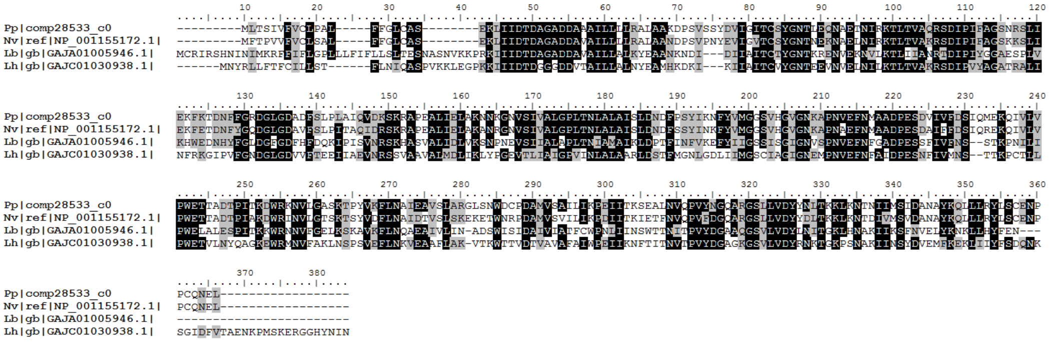

Figure S12. Sequence alignment of inosine-uridine preferring nucleoside hydrolase-like proteins from parasitoid wasp venoms. Pp: *P. puparum*; Nv: *N. vitripennis*; Lb: *L. boulardi*; Lh: *L. heterotoma*.

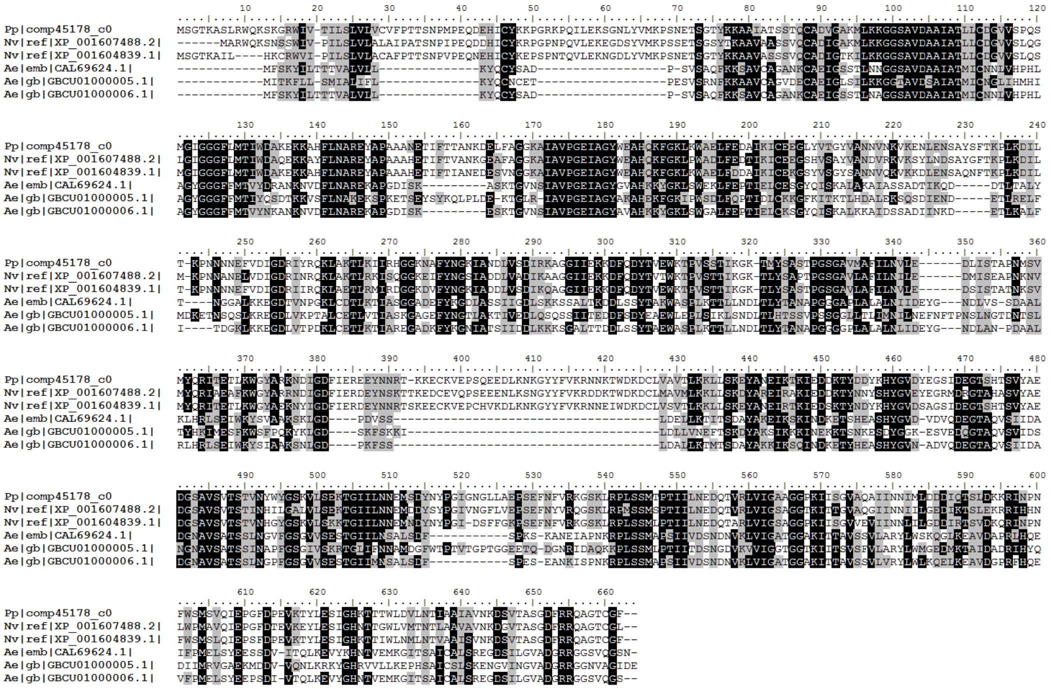

Figure S13. Sequence alignment of gamma-glutamyltranspeptidase from parasitoid wasp venoms. Pp: *P. puparum*; Nv: *N. vitripennis*; Ae: *Aphidius ervi*.

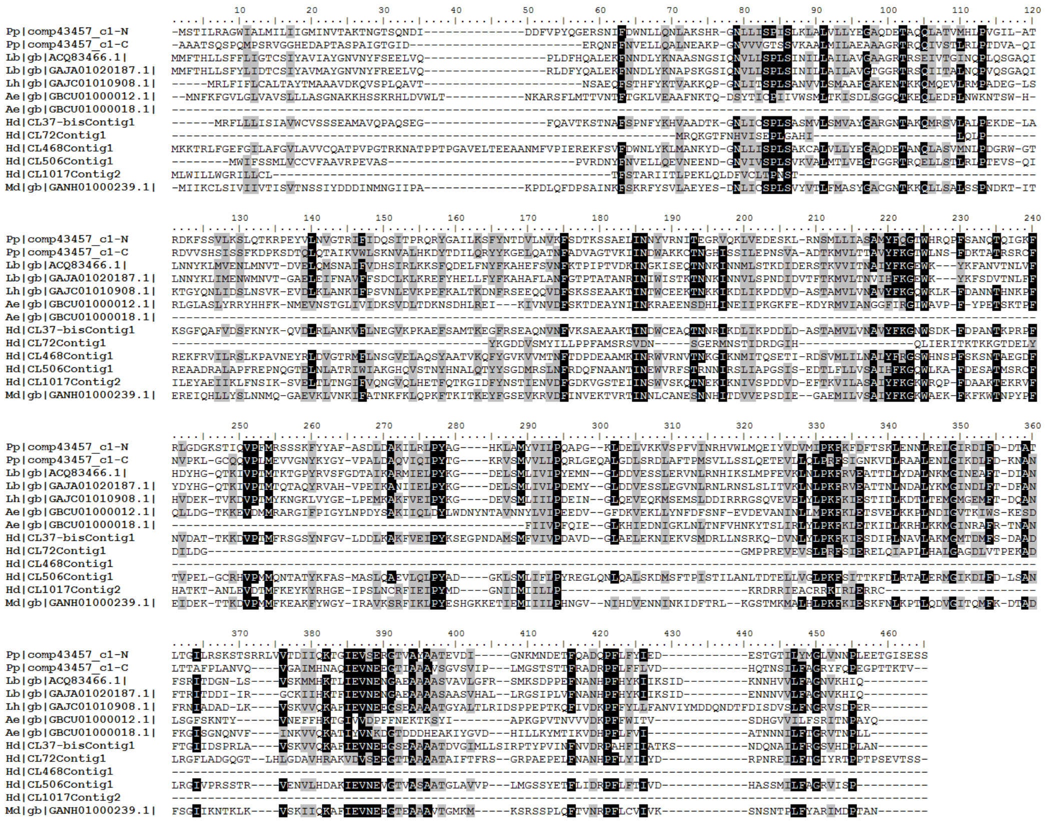

Figure S14. Sequence alignment of serpin from parasitoid wasp venoms. Pp: *P. puparum*; Nv: *N. vitripennis*; Lb: *L. boulardi*; Lh: *L. heterotoma*; Ae: *A. ervi*; Hd: *H. didymator*; Md: *M. demolitor.*
